# The selectivity of galardin and an azasugar-based hydroxamate compound for human matrix metalloproteases and bacterial metalloproteases

**DOI:** 10.1371/journal.pone.0200237

**Published:** 2018-08-03

**Authors:** Ingebrigt Sylte, Rangita Dawadi, Nabin Malla, Susannah von Hofsten, Tra-Mi Nguyen, Ann Iren Solli, Eli Berg, Olayiwola A. Adekoya, Gunbjørg Svineng, Jan-Olof Winberg

**Affiliations:** 1 Department of Medical Biology, Faculty of Health Sciences, UiT-The Arctic University of Norway, Tromsø, Norway; 2 Department of Pharmacy, Faculty of Health Sciences, UiT-The Arctic University of Norway, Tromsø, Norway; George Washington University, UNITED STATES

## Abstract

Inhibitors targeting bacterial enzymes should not interfere with enzymes of the host, and knowledge about structural determinants for selectivity is important for designing inhibitors with a therapeutic potential. We have determined the binding strengths of two hydroxamate compounds, galardin and compound **1b** for the bacterial zinc metalloproteases, thermolysin, pseudolysin and auerolysin, known to be bacterial virulence factors, and the two human zinc metalloproteases MMP-9 and MMP-14. The active sites of the bacterial and human enzymes have huge similarities. In addition, we also studied the enzyme-inhibitor interactions by molecular modelling. The obtained *K*_i_ values of galardin for MMP-9 and MMP-14 and compound **1b** for MMP-9 are approximately ten times lower than previously reported. Compound **1b** binds stronger than galardin to both MMP-9 and MMP-14, and docking studies indicated that the diphenyl ether moiety of compound **1b** obtains more favourable interactions within the S´_1_-subpocket than the 4-methylpentanoyl moiety of galardin. Both compounds bind stronger to MMP-9 than to MMP-14, which appears to be due to a larger S´_1_-subpocket in the former enzyme. Galardin, but not **1b**, inhibits the bacterial enzymes, but the galardin *K*_i_ values were much larger than for the MMPs. The docking indicates that the S´_1_-subpockets of the bacterial proteases are too small to accommodate the diphenyl ether moiety of **1b**, while the 4-methylpentanoyl moiety of galardin enters the pocket. The present study indicates that the size and shape of the ligand structural moiety entering the S´_1_-subpocket is an important determinant for selectivity between the studied MMPs and bacterial MPs.

## Introduction

Proteases are enzymes that cleave peptides and proteins at their N- or C-terminal ends (exopeptidases) or within the polypeptide chain (endopeptidases). They are important for all organisms, and it is estimated that there are more than 66000 different proteases [1, 2]. In microorganisms, proteases are important for generation of nutrition, invasion into host organisms as well as growth and survival [3–9]. In vertebrates, they are involved in the regulation of various physiological processes including cell growth, cell signalling, blood pressure, coagulation, angiogenesis, reproduction, wound repair, hemostasis and homeostasis [2, 10–14]. In humans, diseases are often associated with dysregulation of one or several proteases [10, 11, 15–18] and several proteases are important targets for therapeutic intervention [19–22].

Proteases are divided into classes or clans based on residues involved in the catalytic reaction [2, 23–25]. One of these classes is the metalloproteases (MPs), where the catalytic metal most often is a zinc ion [23, 24]. The bacterial zinc-MPs thermolysin, pseudolysin and auerolysin are secreted by various types of bacteria including bacillus thermoproteolyticus, pseudomonas aeruginosa and staphylococcus aureus, and act as virulence factors [26]. These proteases belong to the M4 family of proteases. Thermolysin is one of the most studied proteases, and has become a model enzyme for the M4 family [26]. In humans, one of the most studied MP families is the matrix metalloproteases (MMPs) also called matrixins [15]. They belong to the M10 family, and in humans there are 23 different MMPs. Seven of them contain a transmembrane domain or a glycosylphosphatidyl-inosityl (GPI) moiety which links them to the cell membrane, while the other sixteen MMPs are secreted enzymes [15]. The most studied among the membrane linked is MT1-MMP (MMP-14), while MMP-9 (gelatinase B) is the most studied among the secreted MMPs [18, 27]. The reason for the intense studies of MMPs is that they are involved in a large variety of physiological processes and that they are dysregulated in a number of different malignant disorders including various types of cancer, arthritis, osteoarthritis, diabetes, cardiovascular-, eye-, brain- and nervous system diseases [15, 18].

Thermolysin, pseudolysin and auerolysin are secreted proteases classified into the clan gluzincins, i.e. their catalytic zinc ion is bound to the protein through two histidines and a glutamate (HEXXH+E) [23, 24, 26]. The fourth zinc ligand is a water molecule that is polarized by the glutamic residue next to the first histidine that binds the catalytic zinc. The catalytic sites of thermolysin and pseudolysin have been extensively studied through X-ray crystallography with various inhibitors bound and several structures are deposited in the protein database (PDB). However, for auerolysin only one X-ray structure of the free enzyme is available.

**Fig 1:**
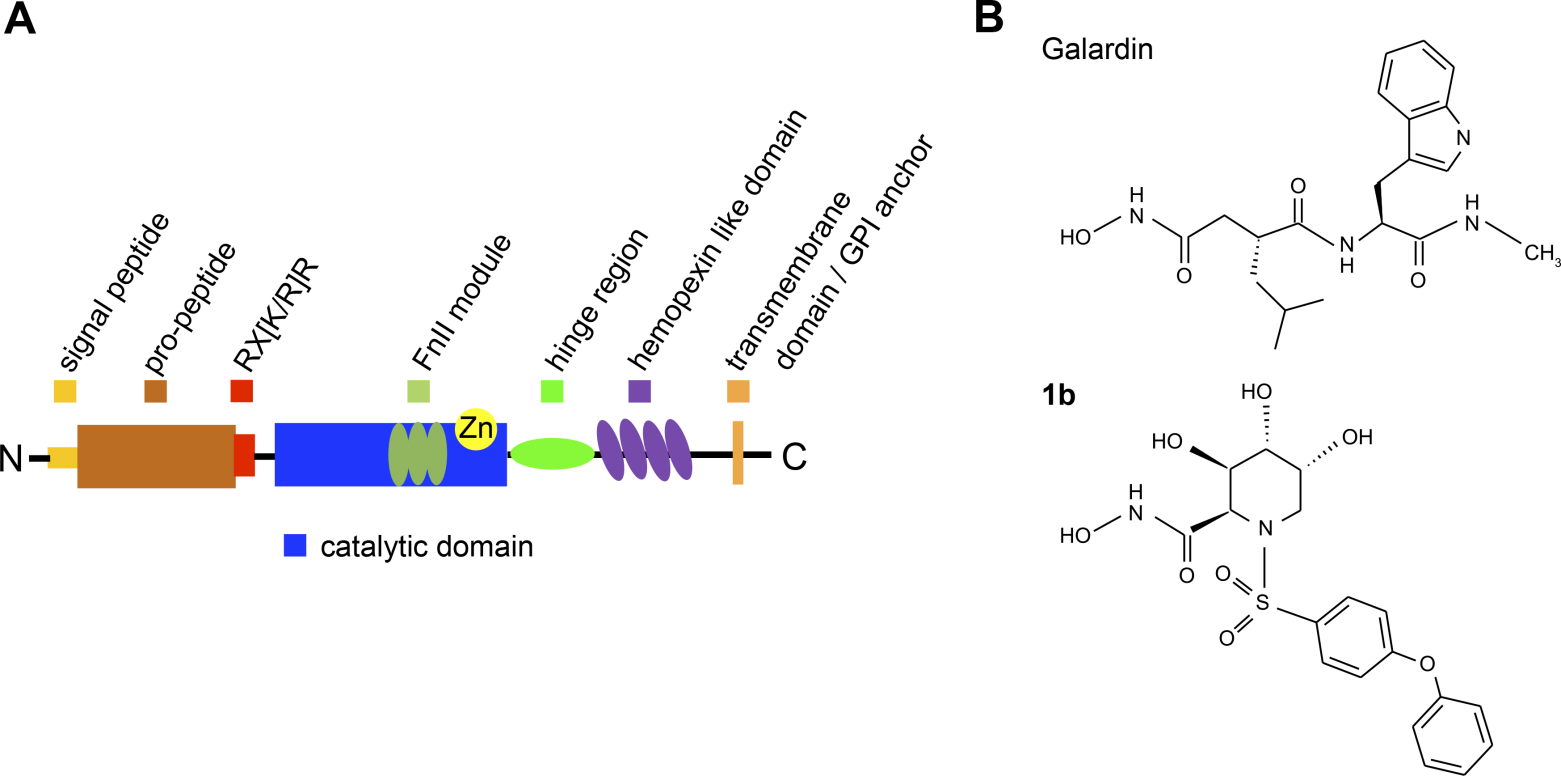
**Domain structure of MMPs (A) and schematic representation of galardin and compound 1b (B).** All MMPs contain a signal peptide (cleaved off in the endoplasmatic reticulum), a pro-peptide domain and a catalytic domain. In addition, most MMPs contain a linker (hinge-region) and a hemopexin (HPX) like domain. The hinge region in MMP-9 differs from the other MMPs as it is longer and heavily O-glycosylated, and therefore also called the OG-domain. Three secreted (MMP-11, -21, -28) and all membrane-anchored MMPs have a basic RX[K/R]R motif at the C-terminal end of their pro-domain. This motif can be cleaved inside the cells by furin-like proteases. The two gelatinases (MMP-2, -9) contain three fibronectin II like repeats (FnII module) in their catalytic domain, located N-terminal to the catalytic Zinc-binding site. Four of the six membrane-type (MT)-MMPs are anchored to the cell membranes through a type I transmembrane domain and the other two through a glycosylphosphatidylinosityl (GPI) moiety.

Like the majority of the MMPs, both MMP-14 and MMP-9 contain an N-terminal signal peptide followed by a pro-domain with the conserved PRCGV sequence to keep the enzyme in a latent state. This is followed by a catalytic domain linked to the C-terminal hemopexin like domain (HPX) through a hinge or linker region which varies in length and structure between the MMPs [15]. The HPX domain in MMPs is involved in complex formation with other biological molecules, activation and substrate specificity [15, 28, 29] and the 3D structure shows that this domain adopts a four bladed β-propeller where blade I is connected to blade IV through a disulphide bridge [30]. Fig 1A shows a schematic drawing of a general MMP structure with its domains and modules. MMPs belong to the clan metzincins and the catalytic zinc ion is bound to the protein through the three histidines of the segment (HEXXHXXGXXH/D+M) [23, 24]. In their inactive pro-form, the fourth zinc ligand is the cysteine of the PRCGV motif of the pro-domain [31, 32]. MMP-14 also contains a type 1 trans-membrane domain C-terminally linked to the HPX domain. At the end of the pro-domain, it contains a basic motif (RX(K/R)R). This motif is recognized by the intracellular serine protease, furin [15]. Hence MMP-14 is activated in the endoplasmatic reticulum and transported to the cell membrane as an active protease lacking its pro-domain, with the active site located in the extracellular environment [15]. MMP-9 on the other hand lacks the (RX(K/R)R) motif and is secreted from cells as an inactive pro-enzyme. This enzyme is unique among the MMPs as it contains a long and heavily O-glycosylated hinge region, also called the OG-domain. In addition, both MMP-9 and MMP-2 contain a large insert in the catalytic site, i.e. three fibronectin II-like motifs (FnII) which is important for the activity against some macromolecular substrates, such as denatured collagen (gelatin) [27, 33–38]. However, the FnII repeats have no effect on the enzymes’ processing of small chromogenic peptide substrates or small inhibitors that only interact with the active site [34]. The long hinge region of MMP-9 is very flexible as shown previously by small angle X-ray crystallography combined with atomic force microscopy and is probably the reason for that the X-ray structure of the full length MMP-9 has not been solved [39]. To what extent the hinge region and the bound sugars, as well as the HPX domain of MMP-9, interact with the catalytic domain is not known. Even though these two domains did not have an effect of MMP-9’s cleavage of some biological macromolecular substrates [40], it cannot be excluded that they are involved in the cleavage of other macromolecular substrates or small chromogenic substrates as well as binding of inhibitors to the catalytic site. MMP-9 can be activated in the extracellular environment by various naturally occurring proteases such as trypsin, kallikrein, MMP-2 and MMP-3. In addition, MMP-9 is also activated by organic mercurial compounds such as p-aminophenylmercuric acetate (APMA) and by bacterial proteases such as thermolysin and pseudolysin [32]. Various activators cleave the MMP-9 pro-domain at different positions resulting in enzyme structures with different N-terminals [32]. In addition, both protease activation and APMA induced auto-activation are also accompanied to various extents with further truncation of the enzyme by cleavage of the HPX domain and in some cases of the OG-domain [41–45]. Binding of inhibitors to the active site of MMP-9 and MMP-14 have been extensively investigated both by kinetic and X-ray crystallography studies [46–54]. For both, inhibitor binding is most often studied by using the recombinant catalytic domains of MMP-14 and MMP-9. In the latter enzyme, the fibronectin II-like module in the catalytic site is lacking in most of the structures deposited in the PDB.

Many bacterial proteases like thermolysin, pseudolysin and auerolysin are virulent factors and hence putative drug targets [5–8, 55]. However, it is important that drugs targeting the bacterial enzymes not interfere with the function of the human MPs. Our focus for some time has been on MP inhibitors [56, 57]. By studying the binding of various inhibitors to bacterial and human MPs, we are aiming to obtain information about similarities and differences in the active site of these enzymes that can be used in the development of compounds that bind specifically to the bacterial enzymes. In the present work we are studying the binding of two hydroxamate containing compounds, galardin and compound **1b** (Fig 1B) to thermolysin, pseudolysin and auerolysin, and to the human MMP-9 and MMP-14. Galardin is a well studied compound that binds strongly to several MMPs including MMP-9 and MMP-14 as well as to thermolysin and pseudolysin [58–60]. Compound **1b** was developed by Moriyama and colleagues [61] and is an aza-sugar based hydroxamate that has been shown to bind strongly to MMP-1, MMP-3, MMP-9 and TACE, but have not been tested against MMP-14 or bacterial MPs. In order to assure an accurate comparison between the enzymes and between compound **1b** and galardin binding, we have thoroughly retested these compounds under the same methodological conditions against thermolysin, pseudolysin, MMP-14 and against differently activated full length MMP-9 isolated from THP-1 cells and recombinant human full length proMMP-9 produced in Sf9 insect cells. The difference between native MMP-9 and recombinant MMP-9 produced in Sf9 insect cells is the extent of glycosylation of the hinge region [40]. As the N-terminal residue of activated MMPs may affect inhibitor binding as previously seen for APMA, MMP-14 and trypsin-activated MMP-2 [62], MMP-9 was activated with three different activators (trypsin, MMP-3 (catalytic domain) and APMA) that give different N-terminal residues. Trypsin and MMP-3 are physiological activators of proMMP-9, and the small amount of TIMP-1 present in the purified proMMP-9 from various cells such as THP-1 has no or limited effects on the trypsin induced activation of proMMP-9, while in contrast the activation induced by other MMPs and APMA will be affected [42, 43, 63]. During mercury poisoning, the presence of mercury ions can result in an uncontrolled activation of proMMPs in the victim. In addition, we have performed molecular modelling studies of the two compounds interaction with the active site of the five enzymes. Comparing binding modes obtained by docking with the experimentally obtained binding strengths increases the understanding of residues and structural motifs important for binding and selectivity.

## Materials and methods

### Materials

TRIS, DMSO and CaCl_2_·2H_2_O and human recombinant MMP-3 catalytic domain were from Merck (Darmstadt, Germany). EDTA and 2-Methoxy-2,4-Diphenyl-3(2H)-Furanone (MDPF) were from Fluka (Buchs, Switzerland). Acrylamide, Commassie Brilliant Blue G-250 and Triton X-100 were from BDH (Poole, UK). RPMI 1640, streptomycin, penicillin, phorbol 12-myristate 13-acetate (PMA), Hepes, Brij-35, SDS, NaCl, p-aminophenylmercuric acetate (APMA), trypsin, soybean trypsin inhibitor (SBTI), Tween-20 and gelatin were purchased from Sigma (St Louis, MO, USA). Magnetic trypsin beads (Mag-Trypsin) were purchased from Takara (Gothenburg, Sweden). Gelatin-Sepharose, Q-Sepharose, Sephadex G-50 (fine), were from GE-Healthcare (Uppsala, Sweden). Unlabelled molecular weight standards were from BioRad (Richmond, CA, USA), while the Spectra^TM^ Multicolor High Range Protein ladder was from Pierce Biotechnology (Rockford, IL, USA). Magic Marker molecular weight standards, NuPAGE Novex 4–12% BisTris gels and Sf9 insect cells were from Invitrogen (Carlsbad, CA, USA). Western Blotting Luminol reagent was from Sancta Cruz (Santa Cruz, CA, USA). Rabbit anti-rat MMP-9 polyclonal antibody (also detect mouse and human MMP-9) was obtained from Chemicon International Inc. (Temecula, CA, USA). HRP-conjugated goat anti-rabbit secondary antibody was from Southern Biotech (Birmingham, AL, USA). Galardin (Gm6001), pseudolysin, thermolysin and recombinant MMP-14 (catalytic domain) were from Calbiochem (San Diego, CA, USA). Spectra^TM^ Mulitcolor High Range Protein Ladder was from Pierce (Rockford, IL, USA). Fetal bovine serum was from Biochrom AG (Berlin, Germany). Auerolysin was from BioCentrum Ltd (Kraków, Poland). The chromogenic substrates Mca-PLGLDpaAR-NH_2_ (ES001) and Mca-RPPGFSAFK(Dnp)-OH (ES005) were from R&D Systems, Inc (Minneapolis, MN, USA). Azasugar-based MMP-inhibitor **1b** was a kind gift from Dr. Hideki Moriyama (Dept. Drug. Disc. Res., Carna Bioscience Inc., Kobe, Japan). Human MMP-9 (recombinant catalytic domain) was from AnaSpec (Fremont, CA, USA). Vivaspin columns with a 10 and 30 kDa cut-off were from Sartorius Stedim Biotech GmbH (Goettingen, Germany). Imperial blue protein stain was from Thermo Scientific (Rockford, Il, USA).

### Production and purification of proMMP-9 from THP-1 cells

The human leukemic monocyte cell-lines THP-1 was a kind gift from Dr. K. Nilsson, Department of Pathology, University of Uppsala, Sweden. The cells were cultured in RPMI 1640 medium with 10% fetal bovine serum, 50 μg/ml of streptomycin, and 100 units/ml of penicillin. To produce proMMP-9, the cells were washed 3 times in serum-free medium and then cultured for 72 h in serum-free RPMI 1640 medium containing 0.1 μM PMA as described earlier [64]. Conditioned medium was harvested, loose cells were pelleted by centrifugation at 1200 rpm (200g) for 10 min. ProMMP-9 was first separated from Chondroitin sulphate proteoglycans (CSPG) and proMMP-9·CSPG heteromers by Q-Sepharose anion exchange chromatography and then purified by Gelatin-Sepharose affinity chromatography as described previously [65].

### Production and purification of recombinant human full length proMMP-9 from Sf9 insect cells

The cDNA encoding human preproMMP-9 (accession number: BC006093.1) cloned into the pReceiver-M02 vector (catalogue number: EX-F0125-M02) was purchased from GeneCopoeia (Rockville, MD). The cDNA was flanked by Invitrogen^TM^ Gateway^TM^ attB-sequences (Invitrogen, Thermo Fisher Scientific Inc.) and was transferred to pDONR221 using Gateway® BP Clonase® II Enzyme mix (Invitrogen, Thermo Fisher Scientific Inc.) and subsequently to BaculoDirect^TM^ Linear DNA (catalogue number: 12362013) using Gateway® LR Clonase® II Enzyme mix preserving the endogenous MMP-9 stop codon. Baculoviruses were produced using Sf9 cells according to the protocol of the BaculoDirect^TM^ Baculovirus Expression System. The P3 viral stock was used for production of preproMMP-9 in Sf9 cells in suspension.

Thirty ml of serum containing medium from baculovirus infected Sf9 cells was applied to a 1 ml column of Gelatin-Sepharose pre-equilibrated with 0.1 M Hepes buffer pH 7.5 containing 5.0 mM CaCl_2_. After collecting the pass-through medium, the column was first washed with 10 column volumes of 0.1 M M Hepes buffer pH 7.5 containing 5.0 mM CaCl_2_ and 1.2 M NaCl. This was followed by a new wash with 30–40 column volumes of 0.1 M Hepes buffer pH 7.5 containing 5.0 mM CaCl_2_. Bound proMMP-9 was eluted with a buffer containing 0.1 M Hepes pH 7.5, 5.0 mM CaCl_2_ and 7.5% DMSO. The eluted material was concentrated and depleted of DMSO (end [DMSO] less than 0.02%) using a spin column with a 10 kDa cut-off. The amount of proMMP-9 in the sample was determined spectrophotometrically at 280 nm using the extinction coefficient ε_280nm_ = 114.36 mM^-1^cm^-1^ [66]. The purified sample was applied to SDS-PAGE (NuPAGE Novex 4–12% Bis-Tris gels). These gels were either further applied to Western blotting (using a polyclonal antibody against proMMP-9) or stained with Imperial blue where bands were cut out and sent to MS analysis at the Tromsø University Proteomics Platform (TUPP). Purified samples were also applied to Gelatin zymography.

### Activation of proMMP-9

Activation of proMMP-9 through treatment of APMA (auto-activation), MMP-3 or trypsin results in a balance between activation and degradation of the enzyme and hence it is important to stop the process when the activation is at its maximum and not to allow the degradation process to go too far.

Activation of proMMP-9 from THP-1 cells was achieved by limited proteolysis with trypsin as described previously, and the activation was stopped by adding soybean trypsin inhibitor (SBTI) [67, 68].

The purified recombinant full length human proMMP-9 from Sf9 cells was activated by 1 mM of APMA at 37°C, MMP-3 (catalytic domain) at 37°C and trypsin covalently linked to magnetic beads (Mag-Trypsin) at room temperature (approximately 23°C). ***Activation with APMA***: Briefly, 55 μl 1.0 mM APMA was added to 500 μl of proMMP-9 (4.6 μM). At various time points, 1.0 μl of this mixture was added to 89 μl assay buffer (0.1 M Hepes pH 7.5 containing 10 mM CaCl_2_, 0.005% Brij-35) and 10 μl 100 μM Mca-PLGLDpaAR-NH_2_. The initial rate of the reaction was determined as described under for the determination of kinetic coefficients. When it was estimated that maximal activation has occurred, the activation was stopped by adding 10 mM EDTA. Thereafter, EDTA and APMA were removed from the activated enzyme by applying the enzyme mixture to a spin column with a 10 kDa cut-off and washed with the assay buffer. ***Activation with Mag-Trypsin***: Briefly, 200 μl Mag-Trypsin was first washed with 5x1 ml of assay buffer and finally 200 μl of the same buffer were added to the beads. Then 200 μl of Mag-Trypsin was mixed with 200 μl of proMMP-9 (4.6 μM), and at various time points 0.5 μl of this mixture was added to 89.5 μl of the assay buffer and 10 μl substrate and the initial rate determined as described above. At the estimated maximal activation, Mag-Trypsin was separated from the active MMP-9 using a strong magnet and the activated MMP-9 was thereafter applied to a spin column with a 30 kDa cut-off and washed with assay buffer resulting in 125000-fold dilution of contaminating peptides. ***Activation with MMP-3 (catalytic domain)***: Briefly, 200 μl proMMP-9 (4.6 μM) was mixed with 200 μl of MMP-3 (0.05 μM) in assay buffer, and at various time points 0.25 μl of the mixture was added to 89.8 μl of assay buffer and 10 μl substrate and the initial rate determined as described above. When it was estimated that maximal activation has occurred, the activation was stopped by adding 10 mM EDTA. Thereafter, the activated MMP-9 was separated from MMP-3 by purification on a Gelatin-Sepharose column as described above, with the exception that the buffer also contained 10 mM EDTA at all washing steps and in the elution step. EDTA and DMSO were removed from the activated enzyme by applying the enzyme mixture to a spin column with a 30 kDa cut-off and washing with the assay buffer. Various dilutions of activated proMMP-9 were used for the detection of kinetic coefficients.

### Gelatin zymography

SDS-substrate PAGE was done as described previously [69] with gels (7.5 cm x 8.5 cm x 0.75 mm) containing 0.1% (w/v) gelatin in both the stacking and separating gel, 4 and 7.5% (w/v) of polyacrylamide, respectively. Gelatinase activity was evident as cleared (unstained) regions.

Real-time gelatin zymography was performed as described previously for standard gelatin zymography [65, 69]. The exception was that 0.1% (w/v) MDPF-fluorescent labelled gelatin was incorporated in the 7.5% SDS-PAGE separating gel instead of 0.1% (w/v) unlabelled gelatin. The fluorescent dye 2-methoxy-2,4-diphenyl-3(2H)-furanone was used to label gelatin to give MDPF-gelatin as described previously [70]. The main difference between normal gelatin zymography and real-time gelatin zymography is that in real-time zymography, the gel is not stained and hence it is possible to follow the degradation of the gelatin in real time without staining. Gelatinase activity was evident as dark bands against the undegraded fluorescent background.

### Western blotting

Purified proMMP-9 from THP-1 cells and recombinant full length human proMMP-9 from Sf9 cells with and without 0.1 M DTT were electrophoresed on SDS-polyacrylamide gel (NuPAGE Novex 4–12% Bis-Tris gels) and electroblotted to a polyvinyl difluoride membrane. After blockage of non-specific binding sites with non-fat milk in TBS-T (150 mM NaCl, 0.25% Tween-20, 20 mM Tris-HCL, pH 7.4), blots were incubated for 1 h at room temperature with primary rabbit polyclonal antibody against human MMP-9. After washing, the blots were incubated for 1 h at room temperature with an HRP-conjugated goat anti-rabbit secondary antibody. The Blots were thereafter washed with TBS-T 3 x 5 min before visualization using Western Blotting Luminol reagent. The intensity of immunoblot bands was measured using a Luminescent Image Analyzer LAS-3000 with MultiGauge software version 3.0 (Fujifilm, Tokyo, Japan).

### Determination of kinetic coefficients

To determine the kinetic and inhibitor kinetic coefficients *K*_m_, and *K*_i_, initial rate experiments were performed using a Perkin Elmer LS 50 Luminescence spectrometer and the FL WinLab Software Package (Perkin Elmer). The reactions were followed for one minute and during that time 600 data points were collected. The excitation and emission wavelengths for the two fluorescence quenched MMP peptide substrates, McaPLGLDpaAR-NH_2_ and Mca-RPPGFSAFK(Dpn)-OH were; λ_ex_ = 320 nm, λ_em_ = 405 nm and a slit width = 10 nm at both wavelengths. All assays were performed at 37°C in an assay buffer of 0.1 M Hepes pH 7.5, 0.005% Brij-35, 10 mM CaCl_2_ and a total assay volume of 100 μl.

#### *K*_m_ determination

Initial rates were determined with McaPLGLDpaAR-NH_2_ and Mca-RPPGFSAFK(Dpn)-OH concentrations ranging from 0.5 to 10.0 μM, higher substrate concentrations resulted in quenching. The *K*_m_ value was calculated from non-linear regression of the Michaelis-Menten equation using the Enzyme kinetic module in GraphPad Prism 5.

#### *K*_i_ determination

A fixed substrate concentration of 5.0 μM and / or 10 μM and a fixed enzyme concentration along with varying concentrations of potential inhibitor were used to determine the inhibitory capacity of the two compounds. From a dose response plot, *v*_i_/*v*_0_ vs the concentration of inhibitor [I], Eq (1) was used to determine the *IC*_50_ values for competitive inhibitors with *K*_i_ > 10 times the concentration of active enzyme in the assay ([E]) where *v*_i_ and *v*_0_ represents the initial rate activity in the presence and absence of inhibitor (I), respectively. For a competitive inhibitor, the *IC*_50_ value equals *K*_i_(1+[S]/*K*_m_). For tight binding competitive inhibitors (*K*_i_ ≤ [E]), the values of *K*_i_ and [E] were obtained by both a dose response plot (*v*_i_/*v*_0_ against [I]) using the Morrison equation [71] (2) and through the linear Henderson Plot [72] (Eq 3). For tight binding inhibitors, the enzyme was pre-incubated for 15 min at 37°C in the presence of inhibitor and the reaction was started by the addition of substrate. Graph Pad Prism 5 was used to calculate *K*_i_ and [E] values.

$$\frac{v_{i}}{v_{0}}=\frac{1}{\left(1+\frac{\left[I\right]}{{\left[IC\right]}_{50}}\right)}$$ (1)

$$\frac{v_{i}}{v_{0}}=1-\frac{\left(\left[E\right]+\left[I\right]+K_{i}\left(1+\frac{\left[S\right]}{K_{m}}\right)\right)-\sqrt{{\left(\left[E\right]+\left[I\right]+K_{i}\left(1+\frac{\left[S\right]}{K_{m}}\right)\right)}^{2}-4\left[E\right]\left[I\right]}}{2\left[E\right]}$$ (2)

$$\frac{\left[I\right]}{\left(1-\frac{v_{i}}{v_{0}}\right)}=K_{i}\left(1+\frac{\left[S\right]}{K_{m}}\right)\left(\frac{v_{0}}{v_{i}}\right)+\left[E\right]$$ (3)

Ninety-six well plates and a Spectra Max Gemini EM Plate Reader controlled by the computer program Soft Max Pro version 4.3 (Molecular Devices) were used to obtain the binding strength of galardin for thermolysin and pseudolysin. Thermolysin (0.22 nM) and psudolysin (0.26 nM) were pre-incubated with galardin for 15 min at 37°C. The final galardin concentrations in the assays varied from 2.42·10^−11^ to 2.42·10^−5^ M. The enzymatic reaction was started by the addition of Mca-RPPGFSAFK(Dpn)-OH (4 μM in assay), and the initial rate of the reaction was followed for 30 min at 37°C using the same wavelengths as with the Perkin Elmer fluorimeter as described above. The *IC*_50_ values were determined from a dose response plot *v*_i_/*v*_0_ vs log [Inhibitor] as described previously [56].

### Docking and scoring

Galardin and compound **1b** were docked using the Internal Coordinate Mechanics (ICM) software version 3.8–4 [73]. The compounds were docked into MMP-9, thermolysin and pseudolysin using several X-ray structures of these enzymes in complex with inhibitor. The following structures in the PDB-database were used: MMP-9; 2ovz, 4xct, 5cuh, 4h3x, thermolysin; 5dpe, 1pe5, pseudolysin; 1u4g, 3dbk. The binding modes of the inhibitors in the X-ray complexes were used to define the binding pocket in the docking process. However, X-ray structure complexes with small molecular inhibitors were not available for MMP-14 and auerolysin. For MMP-14 we used an X-ray structure of the catalytic domain of MMP-14 in complex with the tissue inhibitor of metalloproteinase-2 (TIMP-2) (PDB ID: 1bqq). Two strategies were used for identifying the binding pocket. 1: The MMP-14—TIMP-2 X-ray complex were superimposed with the MMP-9 structure in complex with an inhibitor (PDB ID: 5cuh), and the inhibitor of MMP-9 was used to define the binding pocket in MMP-14. 2: The ICM-pocket finder was used to identify the binding pocket. Both strategies gave similar results. For auerolysin the inhibitor-free X-ray structure (PDB ID: 1bqb) was used for docking. The structure was superimposed with the thermolysin-inhibitor complexes (PDB ID: 5dpe) and (PDB ID: 1pe5) and the inhibitor in these complexes were used to define the binding pocket. Crystallographic water molecules and co-crystallized small molecule inhibitors (MMP-9, thermolysin, pseudolysin) were removed and hydrogen atoms were added and optimized using the ECEPP/3 force field of ICM. Galardin and compound **1b** were built using ICM and minimized before docking. A grid map that included the active site amino acids within 5Å of co-crystallized ligand was calculated, and semi-flexible docking with flexible ligands was performed. Three parallel docking simulations were performed and the best-scored from the parallels was selected as the best orientation. The ICM virtual ligand screening (VLS) scoring function was used for scoring. The compounds were docked both with neutral and charged hydroxamate group.

### Statistical analysis

To compare the obtained *K*_i_ values for the two compounds to the various MPs, a pairwise comparison was obtained by the *t*-test in SigmaPlot (SPSS Corp. Chicago, IL,USA).

## Results and discussion

In the present study, enzyme kinetics and molecular modelling have been used to elucidate the binding of two hydroxamate compounds to the human zinc MPs MMP-9 and MMP-14 and the bacterial zinc MPs thermolysin, pseudolysin and auerolysin. The bacterial enzymes are secreted virulence factors, and inhibitors of these enzymes may weaken the pathogen and be a putative therapeutic strategy against bacterial infections. However, such inhibitors should not interfere with the substrate degradation of MMPs and other endogenous zinc MPs of the infected host. Knowledge about the structural determinants for selectivity is therefore important.

### Synthesis, purification and activation of proMMP-9

**Fig 2:**
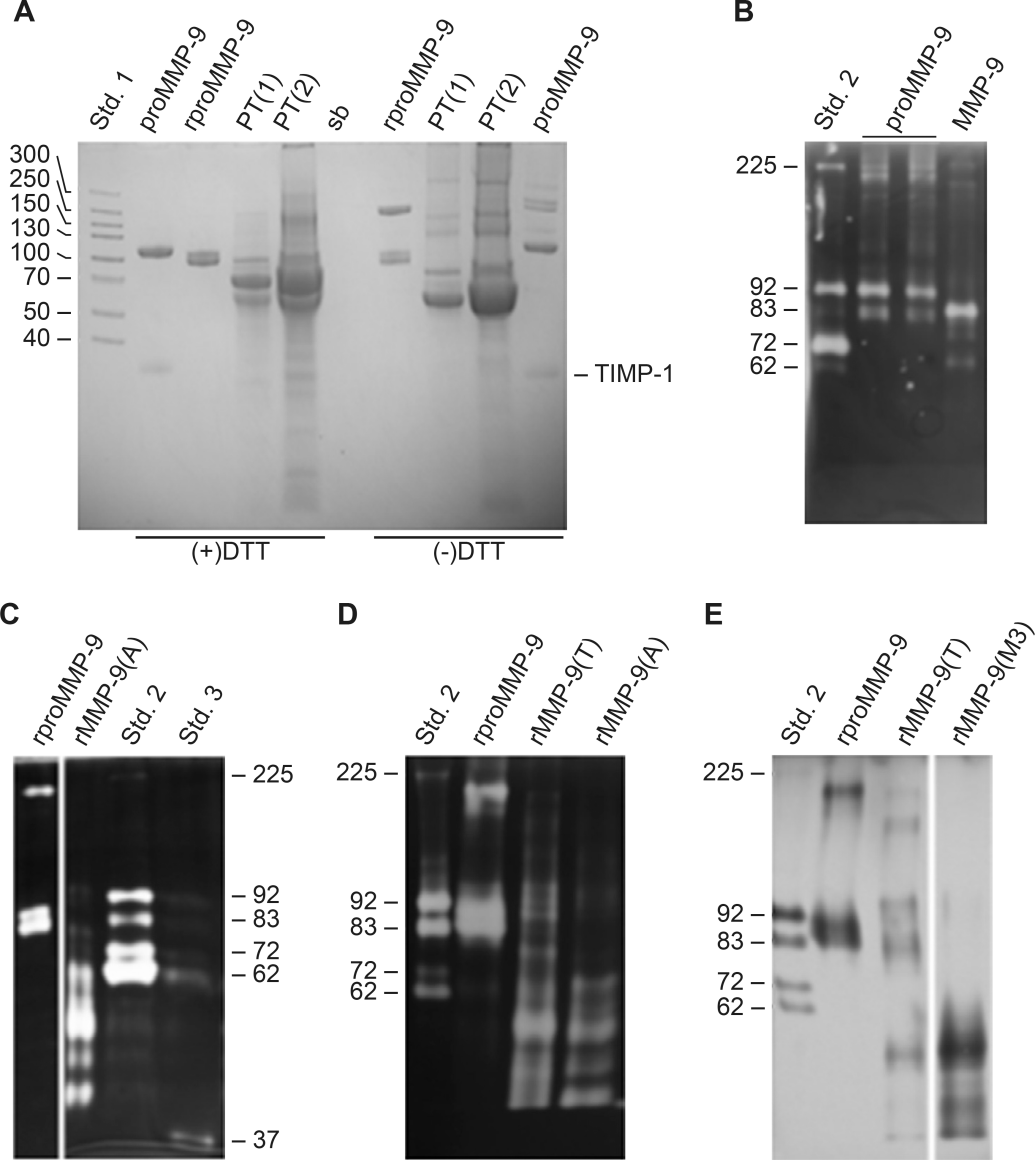
Purification and activation of proMMP-9. (**A**) Imperial stained SDS-PAGE showing the purity of purified recombinant human full length proMMP-9 expressed in Sf9 cells (rproMMP-9) and of proMMP-9 purified from THP-1 cells (proMMP-9) as described in the Materials and Methods section. PT is the pass through fraction from Gelatin-Sepharose Chromatography of the recombinant enzyme, and 4 times more protein was loaded to the gel in the lanes labelled PT(2) compared to the lanes labelled PT(1). Std. 1 is the molecular size marker Spectra^TM^ Mulitcolor High Range Protein Ladder and sb is sample buffer. Prior to electrophoresis, samples were either treated (+) or not treated (-) with DTT. Gelatin (**B-D**) and real-time gelatin (**E**) zymography of purified proMMP-9, trypsin activated (MMP-9) proMMP-9 from THP-1 cells, purified rproMMP-9, AMPA (rMMP-9(A)), trypsin (rMMP-9(T)) and MMP-3 (rMMP-9(M3)) activated recombinant proMMP-9. Std.2 in (**B-E**) is a mixture of proMMP-9 from THP-1 cells and proMMP-2 from human skin fibroblasts. Std. 3 is the 37 kDa catalytic domain of human MMP-9.

ProMMP-9 was purified from conditioned medium of PMA stimulated THP-1 cells by first passing the medium through a Q-Sepharose column to remove chondroitin sulphate proteoglycans (CSPG) and CSPG associated proMMP-9 (proMMP-9∙CSPG) from free proMMP-9 [65]. The pass through fraction from this column contained free proMMP-9, free TIMP-1, TIMP-1 linked to proMMP-9 and other proteins that was applied to a Gelatin-Sepharose column and eluted with 10% DMSO as described earlier [65]. This gave rise to two bands in SDS-PAGE under reducing conditions, a major band at 92 kDa (proMMP-9) and a minor band at 28 kDa (TIMP-1) (Fig 2A). TIMP-1 does not bind to the Gelatin-Sepharose column, but TIMP-1 binds through its C-terminal domain to the C-terminal HPX-domain of proMMP-9 [42, 63, 74, 75] and hence purified proMMP-9 from THP-1 cells will always contain some TIMP-1. The bound TIMP-1 has its N-terminal domain free, the domain that interacts with the active site in MMPs and inhibits the activity. Hence, the presence of proMMP-9·TIMP-1 complexes will affect the activation of proMMP-9 by other active MMPs such as MMP-3. TIMP-1 binds to the active site of these MMPs and form ternary proMMP-9·TIMP-1·MMP and MMP-9·TIMP-1·MMP complexes [63, 74]. The presence of TIMP-1 will not interfere with trypsin during the activation of proMMP-9, but the inhibitor will bind to the active site of the activated MMP-9. TIMPs are slow, tight-binding reversible inhibitors with dissociation constants in the pico-molar region and low dissociation rates of the formed complex [32, 76]. A detailed study of the binding of TIMP-2 to MMP-2 revealed a dissociation constant (*K*_i_) of 0.6 fM and a rate constant for the dissociation of the MMP-2•TIMP-2 complex of 2x10^-8^ s^-1^, i.e. a half-life of approximately 1 year [77]. Similar detailed studies have not been performed for the binding of TIMP-1 to the full length MMP-9, and reliable *K*_i_ values could not be obtained with conventional methods due to the strong binding (low pico-molar region) [78]. The level of MMP-9 activity depends on the amount of TIMP-1 compared to the amount of active MMP-9. With such tight complexes and extremely slow dissociation rates it is fair to assume that the presence of TIMP-1 in the MMP-9 preparation will not affect the *K*_m_ value for a substrate or the *K*_i_ value of an inhibitor compared to an enzyme preparation without TIMP-1 present. Trypsin activation gave rise to a main active form of MMP-9 with a molecular size of approximately 84 kDa, and three minor bands of lower molecular size ranging between 62–80 kDa (Fig 2B). Previously it has been shown that trypsin activated MMP-9 has lost the pro-domain and has F107 (sequence numbering includes the pre-sequence of 19 amino acids) as its N-terminal amino acid residue, and hence the zinc binding motif (97-PRCGVPD) has been removed [32]. The purified proMMP-9 was also treated with 1 mM APMA at 37ºC up to 24h. Zymography revealed that most of the 92 kDa pro-form of MMP-9 had been converted to an 84 kDa form, but no activity could be detected by the rate assay (data not shown). APMA activated MMP-9 has an intact zinc binding motif (97-PRCGVPD) in the pro-domain with M94 (sequence numbering includes the pre-sequence of 19 amino acids) as its N-terminal amino acid residue [32, 41, 43, 45, 79, 80]. The lack of activity is expected, as the enzyme in addition must be C-terminally truncated in order to be active [41, 42, 74].

TIMP-1 free recombinant full length human proMMP-9 was expressed in baculovirus infected Sf9 cells and purified from the serum-containing medium in a one-step procedure using a Gelatin-Sepharose column. This gave rise to three bands in non-reducing SDS-PAGE (Fig 2A) and gelatin zymography (Fig 2C), a band at 205 kDa and two bands at 87 and 83 kDa. SDS-PAGE under reducing and non-reducing conditions (Fig 2A) revealed that the band of 205 kDa was either a homodimer or a homotrimer as recently shown by Vandooren et al. 2015 [81] and Western blotting along with mass spectroscopy (MS) confirmed that all three bands were proMMP-9 (data not shown). The slightly lower molecular size of the recombinant proMMP-9 compared to the native proMMP-9 is most likely due to difference in glycosylation of the hinge region [40]. APMA activation of purified recombinant proMMP-9 in the presence of 10 mM of CaCl_2_ resulted in a main form of active MMP-9 with a molecular size of 54 kDa and three minor forms with molecular sizes of 61, 49 and 45 kDa (Fig 2C). Previously it has been shown that APMA activated MMP-9 has an intact zinc binding motif (97-PRCGVPD) in the pro-domain with M94 (sequence numbering includes the pre-sequence of 19 amino acids) as its N-terminal amino acid residue and parts of the HPX-domain removed [32, 41, 43, 45, 79, 80]. Mercury ion induced auto-cleavage of the MMP-9 HPX domain occurred between E687 and L688 in the end of blade 4 and between Ala526 and Glu527 in the beginning of blade 1 [45]. A disulphide bridge link between C516 and C704 link blade 1 and blade 4 [27, 30]. This suggests that in the 54 kDa active rMMP-9, almost the entire HPX domain is removed, but with the last amino acids in the HPX C-terminal domain (L688—D707) retained linked to the processed enzyme through the disulphide bridge between C516 and C704. Previously it was suggested that the presence of Ca^2+^ resulted in C-terminal truncation and a conformational change that unblocked the catalytic site and hence resulted in an enzyme with full enzymatic activity [79].

Trypsin activation of the purified recombinant proMMP-9 resulted in a zymography pattern similar to the APMA activated enzyme (Fig 2D), with a main form of active MMP-9 with a molecular size slightly larger than 54 kDa. Thus, trypsin induced activation of the recombinant proMMP-9 resulted in both the removal of the N-terminal pro-domain as well as large parts or almost the entire C-terminal HPX-domain similar to the APMA activated enzyme.

As for the trypsin induced activation of the recombinant proMMP-9, MMP-3(catalytic domain) induced activation also resulted in a combination of N- and C-terminal truncation of the recombinant proMMP-9 (S1 Fig). This is in agreement with previous reports on MMP-3 induced activation of proMMP-9 [43, 80]. The fully MMP-3 activated MMP-9 (rMMP-9(M3)) after removal of MMP-3, contaminating peptides, EDTA and DMSO, has a major band at approximately 54 kDa, and two minor bands with lower molecular size (Fig 2E). As for the APMA and trypsin activated MMP-9, the MMP3 activated form also lacks large parts or the entire C-terminal HPX-domain. Previously it was shown that MMP-3 and trypsin-activated MMP-9 has identical N-terminal residue, F107 [32].

### *K*_m_ determination

In order to study the binding strength of inhibitors to an enzyme, it is first necessary to determine the enzyme’s *K*_m_ value for the substrate under the conditions used to study inhibitory binding. The quenched fluorescence substrate Mca-PLGLDpaAR-NH_2_ was used for the studies with MMP-9 and MMP-14. For trypsin activated MMP-9 from THP-1 cells a *K*_m_ value of 3.0 ± 0.7 μM was obtained, while for APMA, trypsin and MMP-3 activated recombinant human MMP-9 (rMMP-9(A), rMMP-9(T) and rMMP-9(M3)) *K*_m_ values of 3.2 ± 0.2, 3.1 ± 0.4 and 4.5 ± 0.4 μM, respectively, were obtained. Thus, there was no significant variation in the obtained *K*_m_ values in spite of differences in their N- and C-terminal amino acid residues. This is in contrast to trypsin and APMA activated MMP-2, where the differences in N- and C-terminal amino acid residues had an effect on the *K*_m_ value for the substrate [62].

The obtained *K*_m_ value for MMP-14 was 6.9 ± 0.9 μM. The *K*_m_ values obtained for MMP-9 and MMP-14 are similar to our previous *K*_m_ values (4 ± 1 μM and 6 ± 1 μM) determined under almost identical conditions, with the exception that the assay in our previous study also contained 5% DMSO [67].

With the bacterial MPs, the quenched fluorescence substrate Mca-RPPGFSAFK(Dnp)-OH was used. The obtained *K*_m_ values for auerolysin, thermolysin and pseudolysin were 47 ± 41 μM, 6 ± 1 μM and 24 ± 8 μM, respectively. For auerolysin and pseudolysin, the *K*_m_ values are far above the highest concentration that could be used in the assay due to quenching at concentrations higher than 10 μM. In spite of the low precision of the obtained *K*_m_ values for auerolysin and pseudolysin, it can be concluded that the substrate concentration of 5.0 μM and 4.0 μM used in the inhibition experiments is far lower than the *K*_m_ value, and hence the obtained *IC*_50_ and *K*_i_^app^ values are close to the real *K*_i_ values.

### *K*_i_ determination

To be able to compare the binding strength of different inhibitors for a given enzyme and of a given inhibitor for different enzymes, it is important that the reported *K*_i_ values are obtained under the same conditions and with the same methods. For tight binding inhibitors, it is necessary to know the amount of active enzyme in the assay in order to obtain a correct *K*_i_ value. As the two inhibitory compounds studied here contain a strong zinc binding residue, the hydroxamate group, the two compounds are believed to be competitive inhibitors. To assure that the obtained *K*_i_ values for the two compounds are as correct as possible and competitive with the substrate, we have varied the concentration of the inhibitors in a series of experiments. Two different concentrations of enzyme have been used, and in some cases also two different substrate concentrations. We have compared the obtained results from two plotting and estimation methods, which determine both the concentration of active enzyme ([E]) in the assay and the *K*_i_ value. One method is using the Morrison equation [71] (Eq 2 in the Materials and Methods section) to fit the results to a plot *v*_i_/*v*_0_ vs [I], while the other is the Henderson plot [72] (Eq 3 in the Materials and Methods section) which is based on a linearized form of the Morrison equation.

#### *K*_i_ MMP-14

**Fig 3:**
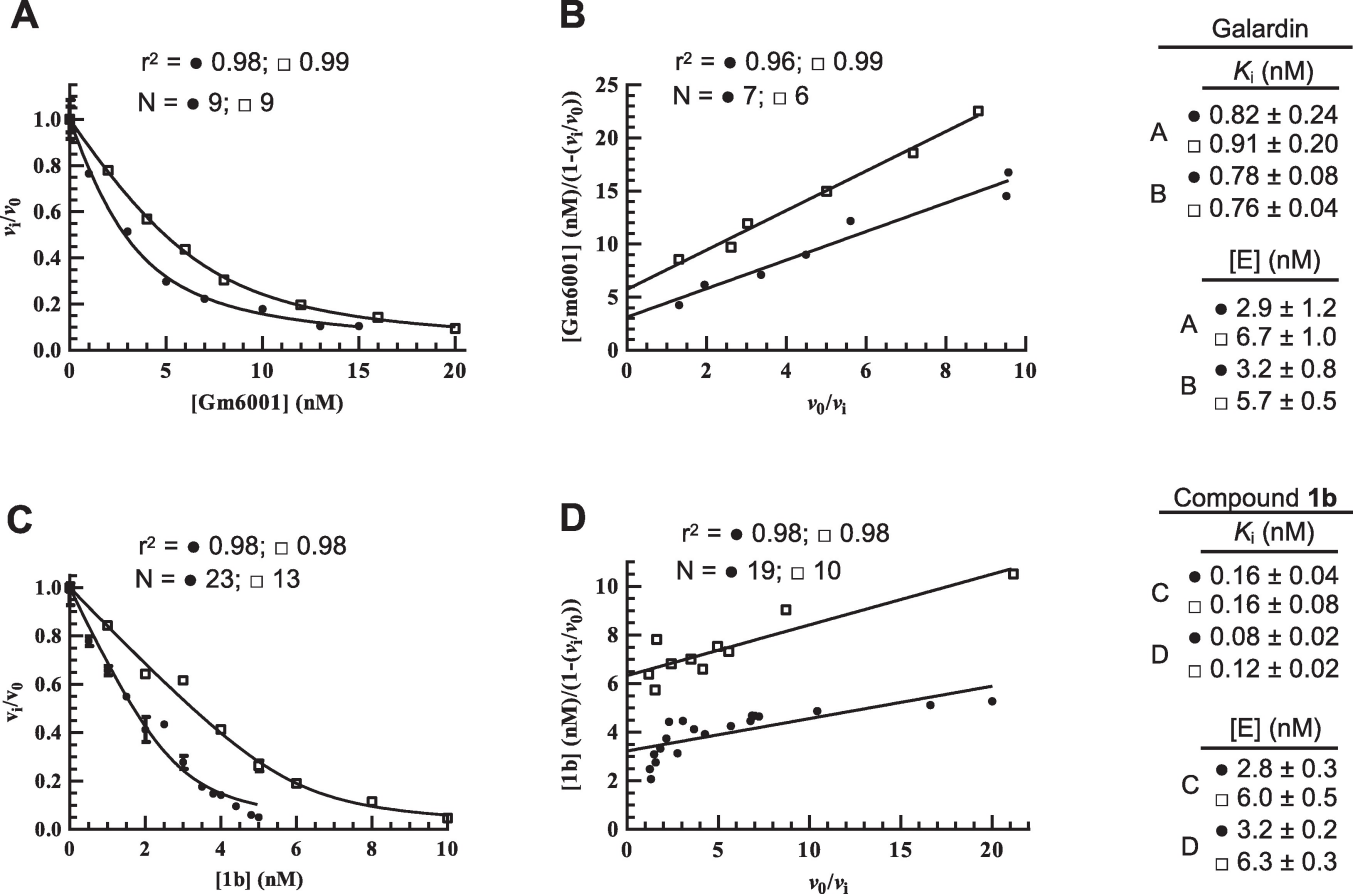
**Inhibition of MMP-14 by galardin (A, B) and compound 1b (C, D).** The inhibition constant *K*_i_ and [MMP-14] in assay were obtained from dose response plots *v*_i_/*v*_0_ vs [I] using the Morrison Eq (2) (**A, C**) and Henderson plots (**B, D**). In all plots, [MMP-14] was twice as high for experiments labelled (□) as for those labelled (●). The [S] is 5.0 μM except in the experiment in (**B**) labelled (□) where it is 10.0 μM. Shown in the figures is also the obtained *K*_i_ and [E] values (mean ± SD), in addition to the regression coefficient r^2^ and the number of individual assays (N) for each curve.

Galardin (Gm6001) inhibits the MMP-14 (catalytic domain) with a reported *IC*_50_ value of 13.4 nM [60], while the *IC*_50_ value is not reported for compound **1b.** Fig 3A shows a typical dose response plot (*v*_i_/*v*_0_ vs [Gm6001]) using two different enzyme concentrations and Fig 3B a typical Henderson plot where one experiment contained twice as high concentration of both MMP-14 and substrate as the other experiment. In the Henderson plot the concentration of MMP-14 in the assay can be directly determined from the regression lines crossing of the y-axis. Furthermore, the slope in a Henderson plot gives an apparent *K*_i_ (*K*_i_^app^) value. For a competitive inhibitor, *K*_i_^app^ equals *K*_i_(1+[S]/*K*_m_) and hence, the slope increases with increasing substrate concentrations. As expected, this is the case for the galardin inhibition of MMP-14 (Fig 3B). Table 1 shows the obtained average *K*_i_ value from the different Henderson plots. Although there were some differences in the results obtained from the Henderson plot and the Morrison equation for an individual experiment (S1 Table and Fig 3), the average *K*_i_ value for galardin was the same, 0.87 nM.

**Table 1:** Inhibitory activity of galardin and compound 1b against human and bacterial metalloproteases.

Protease	*K*_i_ (nM)	
	Galardin	1b
MMP-14	0.870 ± 0.070 (5)	0.090 ± 0.020 (4)
rMMP-9(A)	0.051 ± 0.003 (5)	0.011 ± 0.001 (2)
rMMP-9(T)	0.069 ± 0.001 (5)	N.D.^a^
rMMP-9(M3)	0.063 ± 0.008 (3)	N.D.
MMP-9(T)	0.067 ± 0.006 (4)	0.006 ± 0.000 (2)
Auerolysin	452 ± 35 (1)^b^	N.I.^c^
Thermolysin	20^d^	N.I.
Pseudolysin	20^d^	N.I.

The *K*_i_ values were obtained through Henderson plots as described in materials and methods. Presented is the *K*_i_ ± S.E.M. and in parenthesis the number of independent individual experiments that has given rise to the presented values. The results shown are for recombinant human MMP-14 catalytic domain, recombinant human MMP-9 activated with APMA (rMMP-9(A)), magnetic trypsin beads (rMMP-9 (T)), MMP-3 (rMMP-9(M3), trypsin activated human MMP-9 isolated from THP-1 cells (MMP-9 (T)), auerolysin, thermolysin and pseudolysin.

^a^N.D., not done;

^b^Values from dose response plot using Eq (1) in methods;

^c^N.I., no inhibition up to 100 μM of inhibitor;

^d^Values from Grobelny D et al. [58].

The inhibitory effects of compound **1b** on MMP-14 are shown in Fig 3C and 3D. Although the *K*_i_ values obtained from the Morrison equation are slightly higher than the values obtained from the Henderson plots of the same experiment (S2 Table), it can be concluded that compound **1b** is a significantly stronger MMP-14 binder than galardin (p<0.001; Table 1).

#### *K*_i_ MMP-9

Previous works showed that both galardin and compound **1b** are tight binding inhibitors of MMP-9. The obtained *IC*_50_ and *K*_i_ values for galardin were 0.5 nM and 0.18 nM, respectively [59, 60] and a *K*_i_ value for compound **1b** of 0.097 nM [61]. An MMP may be activated by several different compounds, including various proteases and organo-mercurial compounds like APMA. This often results in removal of non-identical parts from the pro-domain of the given MMP, giving a different N-terminal residue of the activated forms. Differences in N-terminal residue of the activated forms may affect both inhibitor binding and degradation of biological and small chromogenic substrates. Previously we have shown that APMA and trypsin activated MMP-2 have different capacity to bind TIMP-1 as well as different ability to cleave both gelatin and the quenched fluorescence substrate Mca-PLGLDpaAR-NH_2_ [62]. We have therefore tested the binding of galardin to APMA, MMP-3 and trypsin activated MMP-9 and compound **1b** to both APMA and trypsin activated MMP-9. As shown in Tables 1 and S1, galardin binds with similar strength to the four forms of activated MMP-9 (0.051–0.069 nM, Henderson plot; 0.057–0.074 nM, Morrison equation). Furthermore, the *K*_i_ values calculated from the Morrison equation were similar and not statistically different from the values obtained by the Henderson plot (S1 Table). It was only the *K*_i_ value obtained by APMA activated rMMP-9 that was slightly lower than the values for the other activated forms. Disregarding this slight difference in binding strength, it can be concluded that the difference in N- and C-terminal amino acid residues in the different activated rMMP-9 species, the presence of the HPX-domain and small amounts of TIMP-1 (MMP-9) as well as differences in O-glycosylation of the hinge region (rMMP-9 vs MMP-9) have no significant effect on the enzyme’s affinity for galardin.

Compound **1b** binds significantly stronger to both APMA and trypsin activated MMP-9 than galardin (p<0.003; Tables 1, S1 and S2). Furthermore, the *K*_i_ values of compound **1b** calculated from the Morrison equation (0.016 ± 0.001; 0.008 ± 0.003) was not statistically different from the values obtained from the Henderson plots (0.011 ± 0.001; 0.006 ± 0.002) (S2 Table). Furthermore, the difference in *K*_i_ for compound **1b** between rMMP-9 and MMP-9 was not statistically significant (Tables 1 and S2).

#### *K*_i_ bacterial metalloproteases

Previous studies showed that galardin is a strong inhibitor of thermolysin and pseudolysin (Table 1) [58]. In initial scanning experiments of various inhibitors in our laboratory, galardin was used as a control compound. The obtained *K*_i_ values (data not shown) were similar to those obtained by Grobelny et al [58]. Galardin was also a strong inhibitor of auerolysin with a *K*_i_ value much larger than the 3.6 nM of enzyme used in the assay. The line in the Henderson plot crossed at origo. The *K*_i_^app^ value from the slope of the curve was 0.50 ± 0.06 μM (r^2^ = 0.84; N = 16), giving a *K*_i_ value of 0.45 ± 0.05 μM. A dose response plot *v*_i_/*v*_0_ vs [galardin] using Eq (1) in the Materials and Methods section resulted in an *IC*_50_ value of 0.50 ± 0.04 μM (r^2^ = 0.96; N = 18) giving a *K*_i_ value of 0.45 μM (Table 1). Thus, the binding of galardin to auerolysin is about 25 times weaker than the binding to thermolysin and pseudolysin.

Notable, up to the highest concentration tested of compound **1b** (100 μM), no inhibitory effect of the three bacterial MPs was detected (Table 1).

### Docking and molecular modelling

In order to elucidate the structural reasons for the differences in binding affinity for the studied MPs, we have examined the binding modes of galardin and **1b** by docking. Furthermore, it was suggested that the NHOH group of a hydroxamate compound may lose its proton at or close to the physiological pH and generate a negatively charged group (NHO^-^) [82]. Protonation/deprotonation of the NHO(H) moiety may influence the zinc binding properties of hydroxamate compounds, and therefore, **1b** and galardin were docked both with protonated and deprotonated NHO(H) moiety. When available, the compounds were docked into several structures of the enzyme. In that way, target structural flexibility to some extent was taken into account in the docking process. The docking studies indicated that the protonated and deprotonated inhibitors had almost similar binding modes at the active sites. Further, the docking showed that the protonated galardin and **1b** had better docking scores than the deprotonated forms for all enzymes, which is contradictory to a previous docking study of other hydroxamates for the MMP-9, indicating that the deprotonated hydroxamates in general scored better than the protonated [83].

### MMP-9

**Fig 4:**
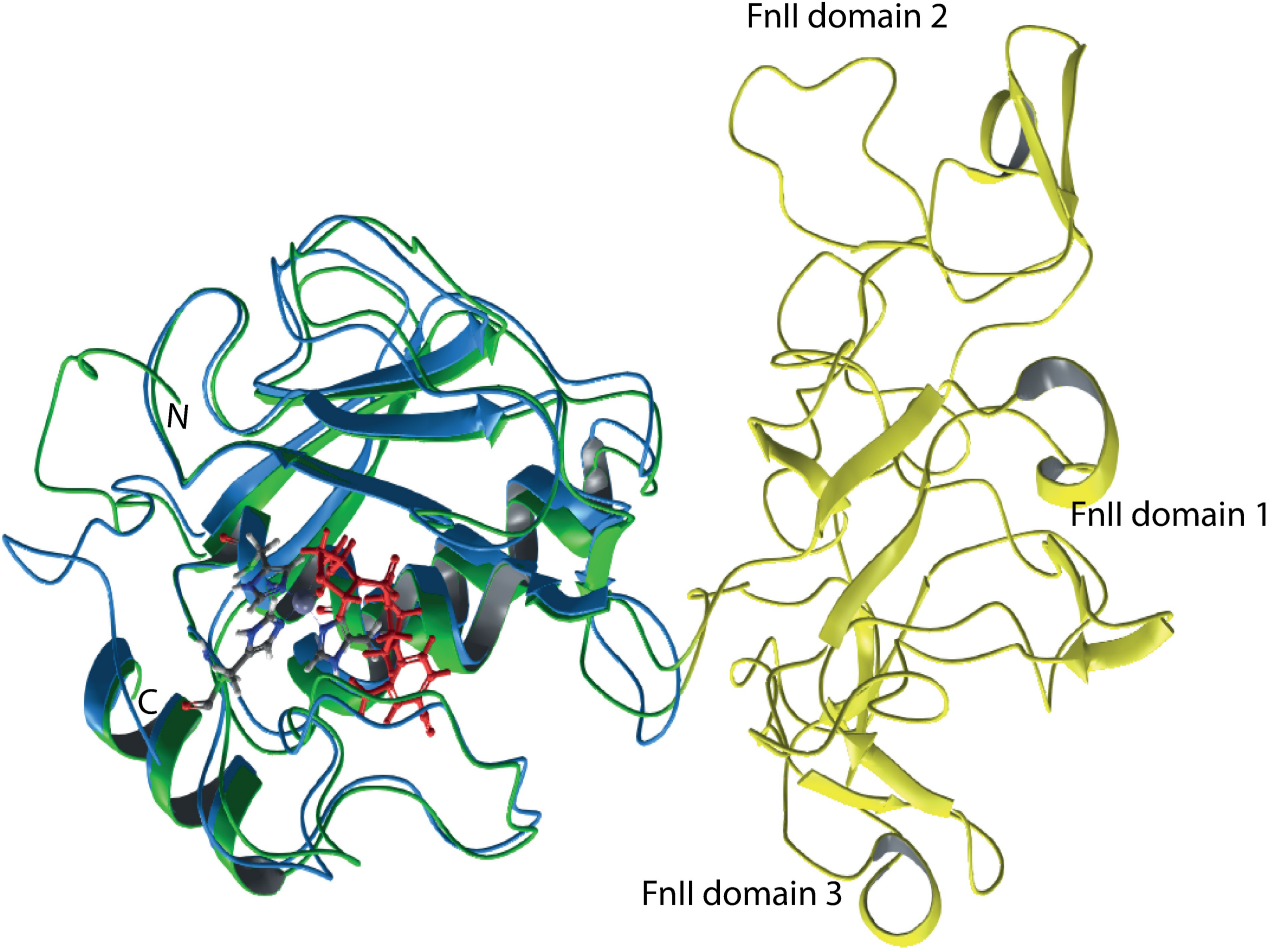
Structural superimposition (backbone) of the 5cuh and the 1l6j x-ray structures of MMP-9. The 1l6j structure contains the FnII domains (yellow) and the catalytic domain (green), while the 5cuh only contains the catalytic domain (blue). The pro-domain has been deleted from 1l6j, such that the sequence starts at F107. The co-crystallized hydroxamate inhibitor LT4 of the 5cuh in red, while the catalytic zinc and the coordinating histidines are in grey. The position of the co-crystallized inhibitor was used to define the docking grid during docking of galardin and compound **1b**, and the figure shows that the docking into a structure lacking the FnII domains (5cuh) should not affect the docking results.

Four MMP-9 complexes from the PDB database were used for docking of both protonated and deprotonated variants of galardin and **1b**. The best scoring values were obtained with the 5cuh (PDB ID) structure. This structure is obtained from a truncated recombinant MMP-9 variant, that is lacking the pro, hinge and C-terminal HPX domains in addition to the FnII module in the catalytic site, and the PDB file is numbered from amino acids G106 to G269. The N-terminal of this recombinant variant is starting at the 106-GFQT segment. The G residue is not present in the natural variant [25, 27] and in trypsin and MMP-3 activated MMP-9 the N-terminal is 107-FQT [32]. In the full-length enzyme, the 175 amino acid long FnII module is present between amino acids V216 and G217 and we therefore used the numbering given in the Merops data base [25] for the amino acids. The X-ray structure of the human proMMP-9 has been resolved at 2.5 Å resolution (PDB id: 1L6J). The structure includes the pro-domain, the catalytic domain and the FnII module. The docking was performed into X-ray complexes with small molecular inhibitors that were used to define the binding region of the inhibitors during docking. A grid map that included all amino acids within 5Å of the co-crystallized inhibitor were used in the docking. The FnII module may influence inhibitor binding, but Fig 4 indicates that the FnII domain is located far from the binding site of small molecular inhibitors, and that the grid map used for docking should not be influenced by the presence of the FnII module. The lack of FnII module in the 5cuh structure should therefore not affect the docking of the molecules in the present study.

**Fig 5:**
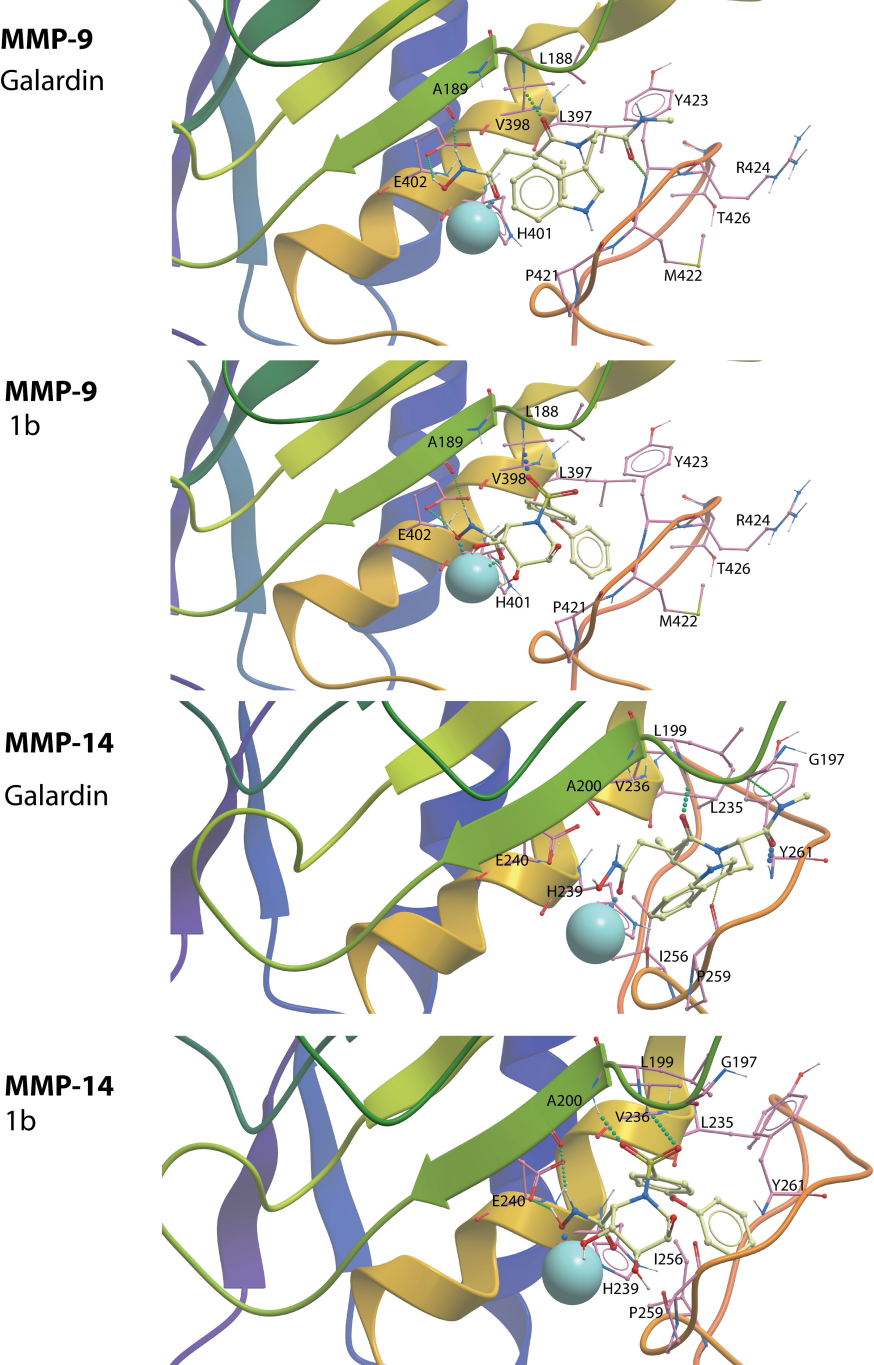
Galardin and compound 1b docked into the catalytic site of MMP-9 and MMP-14. The figure shows close ups of the active site region with the compound structures (xsticks), secondary structure elements and the most important amino acids for ligand binding (xsticks) indicated. Colour coding of atoms of amino acids and ligands: oxygen; red, nitrogen; blue, hydrogen; white, sulphur; yellow, carbon atoms of ligands; yellow, carbon atoms of amino acid side chains; pink, the zinc ion; light blue. The secondary structures elements are coloured from the N- to the C-terminal such that corresponding secondary elements of MMP-9 and MMP-14 obtain similar colour.

**Fig 6:**
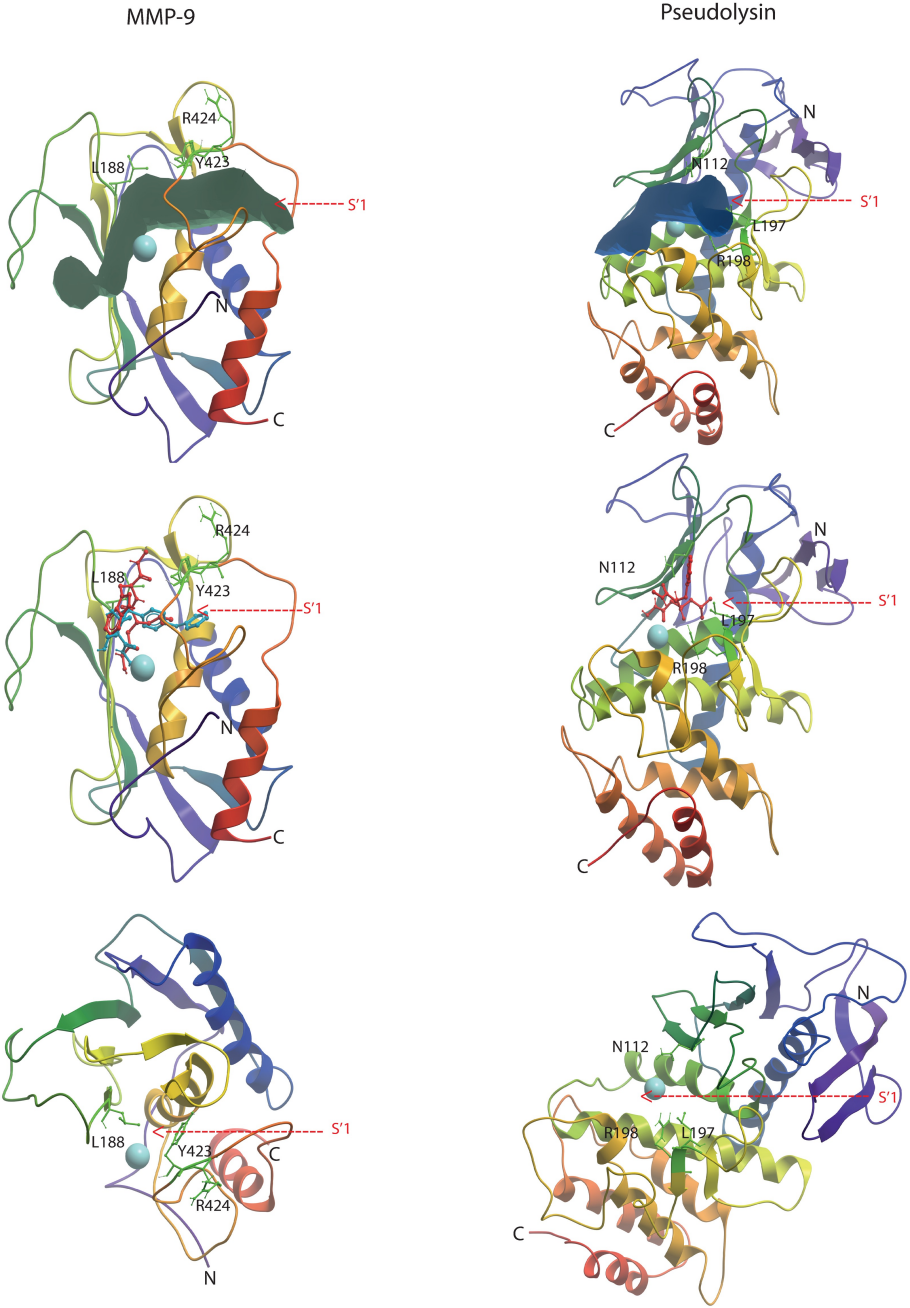
Galardin and compound 1b docked into MMP-9 and galardin docked into pseudolysin. Upper panel: The backbones of MMP-9 (5cuh) and pseudolysin (3dbk). The volume of the full binding pocket identified by the ICM Pocketfinder is displayed for both enzymes, with the S’_1_-subpocket indicated by an arrow. Middle panel: Galardin (red) and compound **1b** (blue) docked into the binding pocket of MMP-9 and galardin (red) docked into pseudolysin. The panel shows that compounds may enter the S’_1_-subpocket of MMP-9 in a region between the side chains of Y423 and L188 on one side and the zinc. The corresponding entrance in pseudolysin is partly hindered by the side chain of R198. Lower panel: The complex from the middle section rotated 90 degrees and the ligands removed. The panel shows that the side chains of L188 and Y423 are located close to each other and hinder the entrance into the S’_1_-subpocket from the region above the zinc, while the corresponding region in pseudolysin is wider (side chains of N112 and L197).

For protonated compound **1b**, both the oxygens on the NHO(H) and CO groups of the hydroxamate moiety binds to the catalytic zinc (Fig 5). In addition, the hydroxamate forms hydrogen bonds between the hydroxyl hydrogen of the NHO(H) moiety and the side chain of E402, while the nitrogen hydrogen of the NHO(H) moiety forms a hydrogen bond with the backbone CO of A189. The heterocyclic ring with its three OH-groups points into the opening of the active site cavity and appears not to have direct interactions with residues of the enzyme. One of the oxygens of the SO_2_ group forms a hydrogen bond with the backbone NH of L188. The diphenyl ether moiety is located within the S´_1_-subpocket (Fig 6) having interactions with the side chains of L188, L397, V398, H401, Y423 and T426 (Figs 5 and 6). Compound **1b** also has van-der Waals interactions with many main chain residues including H401, M422 and R424. In summary, in addition to large van-der Waals interactions, compound **1b** forms 3 hydrogen bonds with the enzyme and 2 ionic interactions with the catalytic zinc ion. The de-protonated form of compound **1b** overlapped with the protonated form and the main difference was that the de-protonated variant lacked the hydrogen bond to the E402 side chain (not shown).

The binding mode of galardin to MMP-9 was similar to that of compound **1b** (Fig 5). The CO and NHO(H) moieties interact with the zinc, E402 and A189 similarly to compound **1b**. The 4-methylpentanoyl moiety in galardin was located in the S´_1_-subpocket, but not as deep into the pocket and with less interaction than the diphenyl ether of compound **1b** (Fig 6). The main interactions of this hydrophobic moiety of galardin are with the side chains of H401, V398, P421 and Y423 (Fig 5). The oxygen of the CO neighbour of the 4-methylpentanoyl moiety and the oxygen on the tryptophan methylamide form hydrogen bonds with the main chain NH of L188 and Y423, respectively. The tryptophan moiety points into the opening of the active site cavity and appears to have no direct interactions with the enzyme. The docking indicates that both protonated **1b** and galardin forms two ionic interactions with the zinc, and three hydrogen bonds with the enzyme. The main difference is that the diphenyl ether moiety of compound **1b** penetrates deeper into the S´_1_-subpocket than the 4-methylpentanoyl moiety of galardin (Fig 6). This appears to be the main explanation for the approximately 5 to 10 times stronger interaction of MMP-9 with compound **1b** than with galardin.

### MMP-14

The docking indicated that compound **1b** and galardin bind MMP-14 in similar binding modes as for MMP-9 (Fig 5). The compounds were docked using two different approaches to define the binding pocket (ICM pocket finder and superimposing with a MMP-9 inhibitor complex), and both approaches gave similar results. Both protonated and un-protonated compounds had quite similar binding modes, however, with some differences.

Protonated **1b** formed more hydrogen bonds with the enzyme than deprotonated **1b** and had better scoring values. In the highest scored binding mode of compound **1b** the two oxygen atoms in the CONHO(H) moiety formed strong interactions with the catalytic zinc. However, due to protonation, the position of the CONHOH is slightly disturbed compared with the deprotonated counterpart, such that the hydroxyl hydrogen of the NHOH forms a hydrogen bond with the side chain of E240, while NH forms a hydrogen bond with the main chain CO of A200 (Fig 5). Such hydrogen bonds are not observed for the deprotonated **1b**. The three OH-groups of the heterocyclic ring point into the opening of the active site cavity without direct interactions with the enzyme. The SO_2_ group of protonated **1b** forms two hydrogen bonds with the main chain NH of residues A200 and L199 (Fig 5), while the SO_2_ of the deprotonated **1b** forms a hydrogen bond with the backbone NH of A200, only. The diphenyl ether moiety is filling up large parts of the S´_1_-subpocket as seen for MMP-9 (Fig 6), having interactions with the side chains of L199, L235, V236, H239, I256, P259 and Y261. The ether oxygen is close to the Nd1 atom of H239. One of the main reasons for the slightly weaker binding of compound **1b** to MMP-14 than to MMP-9 seems to be that the size of the S´_1_-subpocket is larger in MMP-9 (based on calculations by ICM pocket finder) and hence allows for more freedom of the ligand to obtain optimal interactions.

Galardin binds MMP-14 very similar to compound **1b**. The hydroxamate forms two ionic interactions with the catalytic zinc ion, one through the carbonyl oxygen and the other through the oxygen at the NHO(H) group (Fig 5). The hydroxyl hydrogen of the protonated NHO(H) moiety has a hydrogen bond with the side chain of E240. The nitrogen proton at NHO(H) forms a hydrogen bond with the CO of A200. The 4-methylpentanoyl moiety was located in the entrance of S´_1_-subpocket and interacted with the side chains of H239, Y261(Me), L199(cd1) and V236. The amide neighbour of the 4-methylpentanoyl moiety forms two hydrogen bonds, one between the CO oxygen and main chain NH of L199, and the other between the NH and the CO on P259. The methylamide moiety also forms two hydrogen bonds to the main chain of the enzyme, one between the CO group and the NH of Y261 and the other between the NH moiety and the CO of G197. The tryptophan moiety points into the opening of the active site cavity having minimal interactions with the enzyme. The most likely explanation for the 10 times stronger interaction of compound **1b** with the enzyme compared to the interaction with galardin is that the diphenyl ether moiety in the former compound has a larger interaction surface with the S´_1_-subpocket than the 4-methylpentanoyl moiety of galardin.

### Bacterial metalloproteases

**Fig 7:**
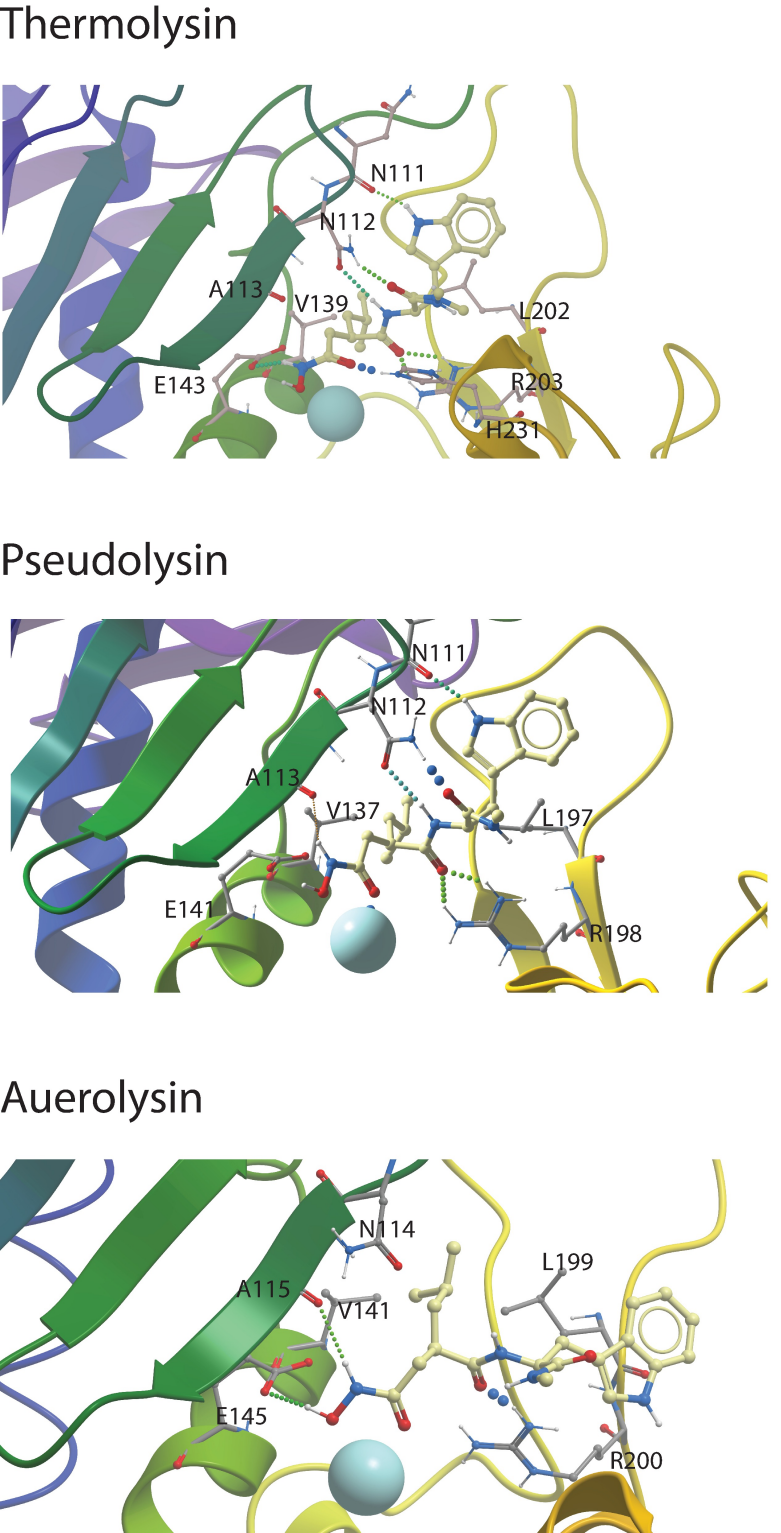
Galardin docked into the catalytic site of thermolysin, pseudolysin and auerolysin. The figure shows close ups of the active site region with the compound structures (xsticks), secondary structure elements and the most important amino acids for ligand binding (xsticks) indicated. Colour coding of atoms of amino acids and ligands: oxygen; red, nitrogen; blue, hydrogen; white, carbon atoms of ligands; yellow, carbon atoms of amino acid side chains; grey, the zinc ion; light blue.

The binding studies indicated that galardin binds quite strongly to thermolysin and pseudolysin and somewhat weaker to aeurolysin, while inhibition of these enzyme by compound **1b** was not observed. These results were also confirmed by the docking studies. Galardin fits into the binding pocket of these enzymes with ionic interactions between the oxygen atoms of the CONHO(H)-moiety and the zinc ion (Fig 7), and with the 4-methylpentanoyl moiety into S´_1_-subpocket (Figs 6 and 7). However, compound **1b,** did not fit into the binding site of the bacterial enzymes, and reasonable binding modes were not obtained in any of the bacterial enzymes. The docking indicates that the diphenyl ether moiety of **1b** is too big for a proper fitting into the S´_1_-subpocket as observed for compound **1b** in the MMPs. The S´_1_-subpocket of the bacterial enzymes seems much more rigid than that of the MMPs. An important reason for that is an arginine (corresponding to R198 of pseudolysin), which is located at the end of a β-strand at the border between the S´_1_- and S´_2_-subpockets and is pointing into the binding site. This arginine interacts with galardin, but does not hinder the smaller 4-methylpentanoyl moiety of galardin to enter the S´_1_-subpocket (Figs 6 and 7), while the diphenyl ether moiety of **1b** is hindered. However, the arginine hinders galardin to penetrate deeply into the pocket. The corresponding region of the studied MMPs constitutes a structurally more flexible loop region (methionine loop) that more easily can adopt to the inhibitor structure.

For thermolysin, the binding mode of protonated and deprotonated galardin were similar. The best scoring was obtained with the X-ray structure 5dpe (PDB ID). The CONHO(H)-moiety forms two ionic interactions with the zinc (Fig 7). The NH group of the hydroxamate is hydrogen bonded with E143 (corresponding to E402 in MMP-9 and E240 in MMP-14), while the CO group is also hydrogen bonded with the side chain of H231 (NH in the ring). The 4-methylpentanoyl was located in the S´_1_-subpocket, while the CO oxygen next to the 4-methylpentanoyl formed two hydrogen bonds with the side chain of R203 (Fig 7). Both the CO and the NH of the tryptophan methylamide are both engaged in hydrogen bonds with the side chain of N112. The NH of the tryptophan ring forms a hydrogen bond with the CO at the backbone of N111.

Most of the interactions of galardin with thermoysin are similar to those in pseudolysin (Fig 7). The amino acids corresponding to E143, H231, R203, N111, and N112 are conserved between the enzymes. The only differences between the highest scored binding mode in thermolysin and pseudolysin was that the NH of the CONHO(H)-moiety was located a bit more distantly from E141 (corresponding to E143 in thermolysin), and that H223 (corresponding to H231 in thermolysin) did not form a hydrogen bond with the CO of the hydroxamate.

The binding mode of galardin with auerolysin (Fig 7) is very similar to the binding modes in thermolysin and pseudolysin, however, there are some differences. Protonated and deprotonated galardin have similar binding modes, but protonated galardin scores better than deprotonated. The CONHO(H)-moiety forms two ionic interactions with zinc, while the protonated OH of the hydroxamate has a hydrogen bond with the side chain of E145 (corresponding to E143 in thermolysin and E141 in pseudolysin). The NH group of the hydroxamate has a hydrogen bond to the backbone CO of A115. The 4-methylpentanoyl is pointing into the S´_1_-subpocket, while the CO oxygen next to the 4-methylpentanoyl forms a hydrogen bond with the side chain of R200 (corresponding to R203 in thermolysin and R198 in pseudolysin). However, the CO and the NH of the tryptophan methylamide are not involved in hydrogen bonding with the enzymes, which may explain the lower binding affinity of galardin for aeurolysin than for thermolysin and pseudolysin.

## Conclusions

Activation of the rproMMP-9 resulted in a largely truncated form in all scenarios, lacking most of or the entire HPX-domain but appearing to contain the entire O-glycosylated hinge region. The main difference between the three activated variants of rMMP-9 seems to be in the N-terminal region. The trypsin and MMP-3 activated forms have F107 and the APMA activated form M94 as the N-terminal residue. The major form of the trypsin activated MMP-9 from THP-1 cells retained its C-terminal HPX-domain due to the presence of some TIMP-1 in the purified enzyme, with F107 as its N-terminal residue. In spite of all these differences, these four differently processed and activated forms had an almost identical *K*_m_ value for the quenched fluorescence substrate Mca-PLGLDpaAR-NH_2_ and *K*_i_ values for galardin and compound **1b**. This suggests that it is possible to compare the average binding strength of the two compounds to each other as well as between the enzymes.

The previously presented binding strength of galardin to MMP-9 and MMP-14 and compound **1b** to MMP-9 appears to be slightly under-estimated compared to the results of the present study. The reason is most likely that these compounds previously have been tested in a series of a large number of compounds against several MPs, and hence detailed kinetic constants were not obtained. An exception is the study of Pourmotabbed et al [59] who studied the binding of galardin to wild-type and mutated un-glycosylated human MMP-9 produced in *E coli*. Both our study and the previous studies show that galardin has a stronger interaction with MMP-9 than with MMP-14. Our study also shows that compound **1b** binds stronger to MMP-9 than to MMP-14 (p<0.002; Tables 1 and S2). An important difference between these two compounds is that compound **1b** binds stronger than galardin to the two human MMPs (Tables 1, S1 and S2), and the docking studies indicated that this could be explained by that the diphenyl ether moiety of compound **1b** has a larger interaction surface with the S´_1_-subpocket than the 4-methylpentanoyl moiety in galardin. Our studies show that galardin binds quite strongly to all the bacterial MPs, but weaker than to the MMPs, while compound **1b** did not bind the bacterial MPs at all. The size and structural rigidity of the S´_1_-subpocket explains why the MMPs more easily adopt to the structure of the inhibitors than do the bacterial MPs. The docking indicated that the diphenyl ether moiety of compound **1b** could not fit into the S´_1_-subpocket of thermolysin, pseudolysin and auerolysin as observed for the MMPs, while the smaller 4-methylpentanoyl moiety of galardin could enter the S´_1_-subpocket. The entrance from the top of the S´_1_-subpocket of the MMPs is quite narrow due to side chain of Y423 and L188 (MMP-9 numbering) as previously explained [56, 57]. However, both galardin and compound **1b** may enter the pocket between the zinc atom and H401 (MMP-9 numbering) on one side and the tyrosine and leucine side chains in the other side. The pocket is also structurally quite flexible since the methionine loop constitutes a large part of the pocket, and therefore the pocket may adopt quite big ligand entities. The entrance from the top of the S´_1_-subpocket of the bacterial enzymes is wider than that of the MMPs. However, an arginine side chain located at the border between the S´_1_ and S´_2_-subpockets is pointing into the binding site and interacts with galardin, and hinders the compound to penetrate deeply into the subpocket (Fig 6). This arginine is located at a rigid β strand, while the corresponding region of the MMPs is in the methionine loop. The docking also indicated that the reason for stronger binding of galardin to thermolysin and pseudolysin than to auerolysin could be explained by hydrogen bonding interactions between the tryptophan methylamide moiety of galardin with two aspargines in pseudolysin and thermolysin (Fig 7). Such interactions were not seen with auerolysin. The present study indicates that the size and shape of the ligand structural moiety entering the S´_1_-subpocket is an important determinant for selectivity between the studied MMPs and bacterial MPs. Compounds with less interaction with the S´_1_- subpocket, but occupy other subpockets may bind more selectively to the bacterial enzymes than to the MMPs.

## Supporting information

S1 Fig Activation of full length recombinant human proMMP-9 with MMP-3(catalytic domain). **(A)** Twenty µL of 4.6 µM proMMP-9 was mixed with 20 µL of 0.05 µM MMP-3 at 37°C. At different time points either 0.5 µL (up to 30 min) or 0.25 µL (from 40 min) of activation mixture was added to 99 µL of 10 µM substrate (in assay buffer) and the enzyme activity (initial rate) was determined as described in methods. **(B)** At the same time points as in (A), 0.5 µL of activation mixture was mixed with 19.5 µL of 10 mM EDTA (in assay buffer). This mixture was further diluted (12.5 times) and mixed with sample buffer and applied to real-time gelatin zymography as described in methods. The molecular size standards used were proMMP-9 purified from THP-1 cells (proMMP-9), recombinant human full length proMMP-9 purified (rproMMP-9) from sf9 cells, the 37 kDa catalytic domain of MMP-9 (Std 3) and a mixture of proMMP-9 from THP-1 cells and proMMP-2 from human skin fibroblasts (St 2).
(PDF)

S1 TableInhibitory constant *K*_i_ of galardin against human metalloproteases.The *K*_i_ ± s.d. values for each experiment were obtained through both Henderson plots and the Morrison equation as described in materials and methods. Shown is also the average $\overset{-}{x}$ ± S.E.M. value for each enzyme and plot. The results shown are for recombinant human MMP-14 catalytic domain, recombinant human MMP-9 activated with APMA (rMMP-9(A)), magnetic trypsin beads (rMMP-9 (T)), MMP-3 (rMMP-9(M3) and trypsin activated human MMP-9 isolated from THP-1 cells (MMP-9 (T)).(PDF)

S2 TableInhibitory constant *K*_i_ of compound 1b against human metalloproteases.The *K*_i_ ± s.d. values for each experiment were obtained through both Henderson plots and the Morrison equation as described in materials and methods. The average $\overset{-}{x}$ ± S.E.M. values for each enzyme and plot are also shown. The results shown are for recombinant human MMP-14 catalytic domain, recombinant human MMP-9 activated with APMA (rMMP-9(A)) and trypsin activated human MMP-9 isolated from THP-1 cells (MMP-9(T)).(PDF)
