# Supplementary material for: The selectivity of galardin and an azasugar-based hydroxamate compound for human matrix metalloproteases and bacterial metalloproteases
Source: PLoS One. 2018 Aug 3;13(8):e0200237. doi: 10.1371/journal.pone.0200237 (PMC6075749; doi:10.1371/journal.pone.0200237)
Supplement: S1 Fig — (A) Twenty µL of 4.6 µM proMMP-9 was mixed with 20 µL of 0.05 µM MMP-3 at 37°C. At different time points either 0.5 µL (up to 30 min) or 0.25 µL (from 40 min) of activation mixture was added to 99 µL of 10 µM substrate (in assay buffer) and the enzyme activity (initial rate) was determined as described in methods. (B) At the same time points as in (A), 0.5 µL of activation mixture was mixed with 19.5 µL of 10 mM EDTA (in assay buffer). This mixture was further diluted (12.5 times) and mixed with sample buffer and applied to real-time gelatin zymography as described in methods. The molecular size standards used were proMMP-9 purified from THP-1 cells (proMMP-9), recombinant human full length proMMP-9 purified (rproMMP-9) from sf9 cells, the 37 kDa catalytic domain of MMP-9 (Std 3) and a mixture of proMMP-9 from THP-1 cells and proMMP-2 from human skin fibroblasts (St 2). (PDF) [file pone.0200237.s001.pdf]

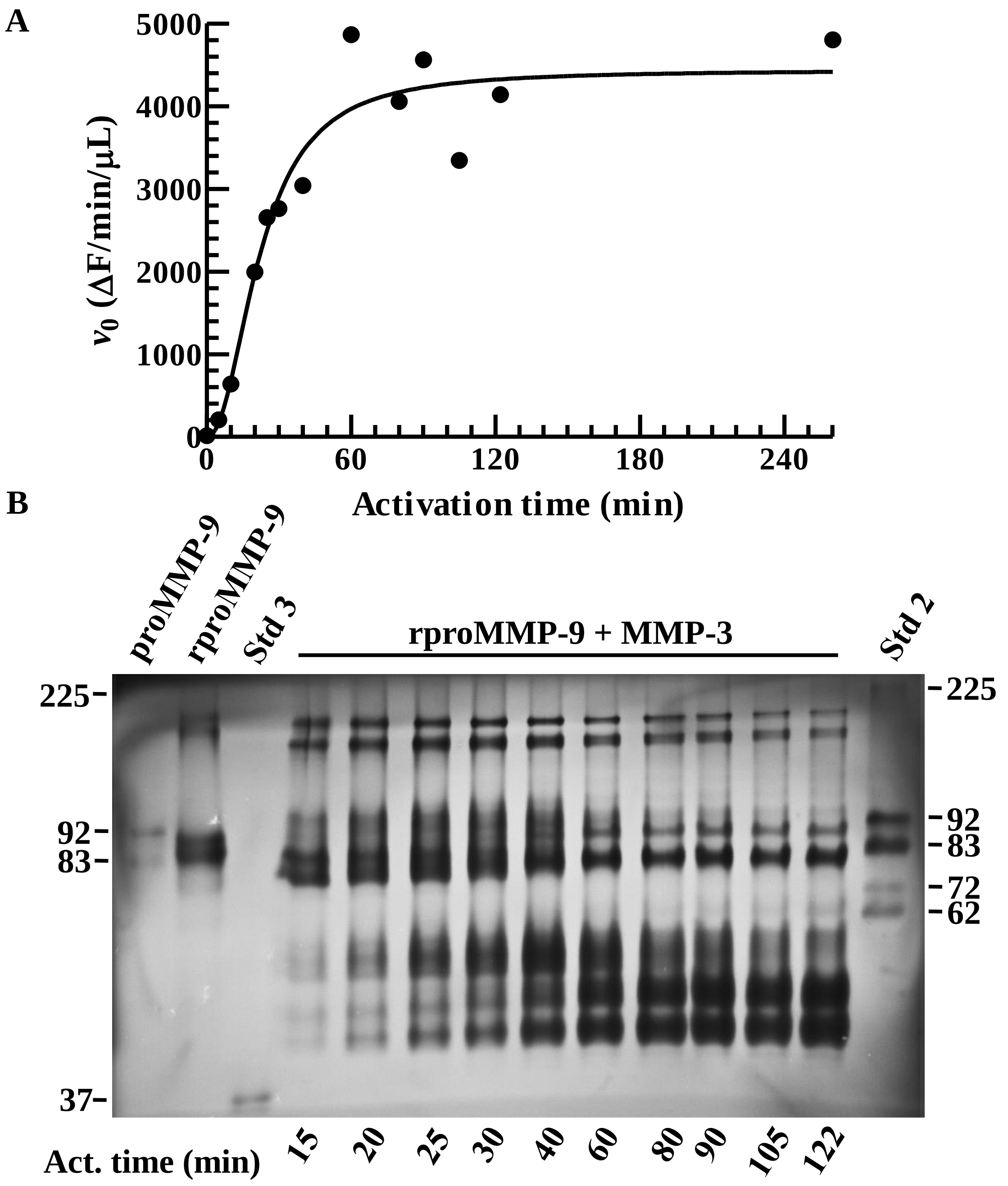

S1 Fig. Activation of full length recombinant human proMMP-9 with MMP-3(catalytic domain). **(A)** Twenty µL of 4.6 µM proMMP-9 was mixed with 20 µL of 0.05 µM MMP-3 at 37 °C. At different time points either 0.5 µL (up to 30 min) or 0.25 µL (from 40 min) of activation mixture was added to 99 µL of 10 µM substrate (in assay buffer) and the enzyme activity (initial rate) was determined as described in methods. **(B)** At the same time points as in (A), 0.5 µL of activation mixture was mixed with 19.5 µL of 10 mM EDTA (in assay buffer). This mixture was further diluted (12.5 times) and mixed with sample buffer and applied to real-time gelatin zymography as described in methods. The molecular size standards used were proMMP-9 purified from THP-1 cells (proMMP-9), recombinant human full length proMMP-9 purified (rproMMP-9) from sf9 cells, the 37 kDa catalytic domain of MMP-9 (Std 3) and a mixture of proMMP-9 from THP-1 cells and proMMP-2 from human skin fibroblasts (St 2).
