# Supplementary material for: The selectivity of galardin and an azasugar-based hydroxamate compound for human matrix metalloproteases and bacterial metalloproteases
Source: PLoS One. 2018 Aug 3;13(8):e0200237. doi: 10.1371/journal.pone.0200237 (PMC6075749; doi:10.1371/journal.pone.0200237)
Supplement: S1 Table — The Ki ± s.d. values for each experiment were obtained through both Henderson plots and the Morrison equation as described in materials and methods. Shown is also the average x- ± S.E.M. value for each enzyme and plot. The results shown are for recombinant human MMP-14 catalytic domain, recombinant human MMP-9 activated with APMA (rMMP-9(A)), magnetic trypsin beads (rMMP-9 (T)), MMP-3 (rMMP-9(M3) and trypsin activated human MMP-9 isolated from THP-1 cells (MMP-9 (T)). (PDF) [file pone.0200237.s002.pdf]

S1 Table. Inhibitory constant K_i of galardin against human metalloproteases.

Protease	Experiment	Galardin K_i (nM) Henderson Plot	Galardin K_i (nM) Morrison equation
MMP-14	1	0.78 ± 0.08	0.82 ± 0.24
	2	0.86 ± 0.05	0.91 ± 0.20
	3	0.76 ± 0.04	0.89 ± 0.27
	4	0.78 ± 0.05	0.95 ± 0.15
	5	1.16 ± 0.04	0.78 ± 0.13
	$\bar{x}$ ± S.E.M.	0.87 ± 0.07	0.87 ± 0.03
rMMP-9 (A)	1	0.046 ± 0.002	0.053 ± 0.004
	2	0.061 ± 0.011	0.053 ± 0.012
	3	0.045 ± 0.010	0.044 ± 0.002
	4	0.051 ± 0.010	0.064 ± 0.020
	5	0.053 ± 0.012	0.070 ± 0.019
	$\bar{x}$ ± S.E.M.	0.051 ± 0.003	0.057 ± 0.005
rMMP-9 (T)	1	0.065 ± 0.013	0.068 ± 0.022
	2	0.068 ± 0.004	0.067 ± 0.005
	3	0.071 ± 0.005	0.081 ± 0.009
	4	0.071 ± 0.016	0.062 ± 0.022
	5	0.070 ± 0.010	0.075 ± 0.016
	$\bar{x}$ ± S.E.M.	0.069 ± 0.001	0.071 ± 0.003
rMMP-9 (M3)	1	0.057 ± 0.004	0.076 ± 0.008
	2	0.054 ± 0.003	0.058 ± 0.004
	3	0.078 ± 0.008	0.088 ± 0.005
	$\bar{x}$ ± S.E.M.	0.063 ± 0.008	0.074 ± 0.009
MMP-9 (T)	1	0.075 ± 0.005	0.078 ± 0.007
	2	0.075 ± 0.010	0.074 ± 0.027
	3	0.065 ± 0.004	0.075 ± 0.009
	4	0.051 ± 0.001	0.067 ± 0.009
	$\bar{x}$ ± S.E.M.	0.067 ± 0.006	0.074 ± 0.002

The K_i ± s.d. values for each experiment were obtained through both Henderson plots and the Morrison equation as described in materials and methods. Shown is also the average $\bar{x}$ ± S.E.M. value for each enzyme and plot. The results shown are for recombinant human MMP-14 catalytic domain, recombinant human MMP-9 activated with APMA (rMMP-9(A)), magnetic trypsin beads (rMMP-9 (T)), MMP-3 (rMMP-9(M3) and trypsin activated human MMP-9 isolated from THP-1 cells (MMP-9 (T)).
