# Supplementary material for: The selectivity of galardin and an azasugar-based hydroxamate compound for human matrix metalloproteases and bacterial metalloproteases
Source: PLoS One. 2018 Aug 3;13(8):e0200237. doi: 10.1371/journal.pone.0200237 (PMC6075749; doi:10.1371/journal.pone.0200237)
Supplement: S2 Table — The Ki ± s.d. values for each experiment were obtained through both Henderson plots and the Morrison equation as described in materials and methods. The average x- ± S.E.M. values for each enzyme and plot are also shown. The results shown are for recombinant human MMP-14 catalytic domain, recombinant human MMP-9 activated with APMA (rMMP-9(A)) and trypsin activated human MMP-9 isolated from THP-1 cells (MMP-9(T)). (PDF) [file pone.0200237.s003.pdf]

S2 Table. Inhibitory constant K_i of compound 1b against human metalloproteases.

Protease	Experiment	**1b** K_i (nM) Henderson Plot	**1b** K_i (nM) Morrison equation
MMP-14	1	0.074 ± 0.015	0.16 ± 0.04
	2	0.128 ± 0.023	0.16 ± 0.08
	3	0.081 ± 0.029	0.10 ± 0.06
	4	0.070 ± 0.047	0.21 ± 0.12
	$\bar{x}$ ± S.E.M.	0.088 ± 0.015	0.16 ± 0.02
rMMP-9 (A)	1	0.010 ± 0.006	0.015 ± 0.009
	2	0.012 ± 0.006	0.017 ± 0.013
	$\bar{x}$ ± S.E.M.	0.011 ± 0.001	0.016 ± 0.001
MMP-9 (T)	1	0.006 ± 0.002	0.006 ± 0.002
	2	0.006 ± 0.002	0.010 ± 0.004
	$\bar{x}$ ± S.E.M.	0.006 ± 0.000	0.008 ± 0.002

The K_i ± s.d. values for each experiment were obtained through both Henderson plots and the Morrison equation as described in materials and methods. The average $\bar{x}$ ± S.E.M. values for each enzyme and plot are also shown. The results shown are for recombinant human MMP-14 catalytic domain, recombinant human MMP-9 activated with APMA (rMMP-9(A)) and trypsin activated human MMP-9 isolated from THP-1 cells (MMP-9(T)).
